# HLA Class I Binding of HBZ Determines Outcome in HTLV-1 Infection

**DOI:** 10.1371/journal.ppat.1001117

**Published:** 2010-09-23

**Authors:** Aidan MacNamara, Aileen Rowan, Silva Hilburn, Ulrich Kadolsky, Hiroshi Fujiwara, Koichiro Suemori, Masaki Yasukawa, Graham Taylor, Charles R. M. Bangham, Becca Asquith

**Affiliations:** 1 Department of Immunology, Faculty of Medicine, Imperial College, London, United Kingdom; 2 Section of Infectious Diseases, Faculty of Medicine, Imperial College, London, United Kingdom; 3 Department of Bioregulatory Medicine, Graduate School of Medicine, Ehime University, and Ehime University Proteomedicine Research Center, Toh-on city, Ehime, Japan; Fred Hutchinson Cancer Research Center, United States of America

## Abstract

CD8^+^ T cells can exert both protective and harmful effects on the virus-infected host. However, there is no systematic method to identify the attributes of a protective CD8^+^ T cell response. Here, we combine theory and experiment to identify and quantify the contribution of all HLA class I alleles to host protection against infection with a given pathogen. In 432 HTLV-1-infected individuals we show that individuals with HLA class I alleles that strongly bind the HTLV-1 protein HBZ had a lower proviral load and were more likely to be asymptomatic. We also show that in general, across all HTLV-1 proteins, CD8^+^ T cell effectiveness is strongly determined by protein specificity and produce a ranked list of the proteins targeted by the most effective CD8^+^ T cell response through to the least effective CD8^+^ T cell response. We conclude that CD8^+^ T cells play an important role in the control of HTLV-1 and that CD8^+^ cells specific to HBZ, not the immunodominant protein Tax, are the most effective. We suggest that HBZ plays a central role in HTLV-1 persistence. This approach is applicable to all pathogens, even where data are sparse, to identify simultaneously the HLA Class I alleles and the epitopes responsible for a protective CD8^+^ T cell response.

## Author Summary

A large immune response to the retrovirus HTLV-1 does not always prevent HTLV-1-associated diseases. Indeed, it has been shown that CD8^+^ T cells may contribute towards the inflammatory disease associated with HTLV-1 infection. This observation has led to the hypothesis that it is the ‘quality’ of the immune response towards HTLV-1 that is important, and not simply a response in itself. Using a combination of computational and experimental methods we have investigated T cell ‘quality’. We have found that specificity is an important determinant of CD8^+^ T cell quality with recognition of the viral protein HBZ enabling the host to make a more effective immune response. This approach can be used for other pathogens to identify what HLA class 1 alleles and the parts of the pathogen they bind to are responsible for a protective CD8^+^ T cell immune response. This work informs basic immunology: “what constitutes a protective CD8^+^ T cell response?”; vaccine design: “which antigens elicit the most effective response” and virology: “which viral proteins are key players in the strategy of persistence?”.

## Introduction

Human T cell lymphotropic virus-type 1 (HTLV-1) is an oncogenic retrovirus that infects between 10 and 20 million people worldwide. Of these infected individuals, 1–6% develop adult T cell leukaemia/lymphoma (ATL/ATLL) and a further 2 to 3% develop a variety of chronic inflammatory syndromes including HTLV-1-associated myelopathy/tropical spastic paraparesis (HAM/TSP); the rest remain lifelong asymptomatic carriers (ACs) of the virus.

Most HTLV-1-infected individuals mount a large, chronically activated CD8^+^ T cell response to HTLV-1 and it is unclear why this fails to eradicate the virus. Furthermore, there is evidence for both protective [1]–[3] and pathogenic effects [4]–[7] of HTLV-1-specific CD8^+^ T cells. The attributes of a protective anti-HTLV-1 response *in vivo* are unknown, although specificity for the viral protein Tax is a strong candidate. There are good reasons to believe that a Tax-specific CD8^+^ response [8] may be particularly protective. Firstly, Tax is the immunodominant HTLV-1 antigen [9], [10]. Secondly, HLA-A*02, which is associated with protection in southern Japan [11], binds several Tax epitopes [12], notably Tax 11–19, which is bound unusually strongly [13]. Thirdly, Tax is one of the first HTLV-1 proteins to be expressed and it has been shown, for HIV-1-infected cells *in vitro*, that CD8^+^ T cells specific to early viral proteins are particularly effective in viral control [14]. Finally, it has been shown that the selective pressure exerted on Tax is higher in asymptomatic carriers than in those that have developed HAM/TSP [15].

What constitutes an effective CD8^+^ T cell response is difficult to ascertain in any infection. Measurements of CD8^+^ T cell frequency, phenotype, function and specificity are informative but, because antigen load influences each of these factors, it can be difficult to ascertain if a particular immune profile is the cause or effect of good pathogen control [16]–[19]. An alternative approach is host genotype analysis. Polymorphisms in immune-related genes, particularly the HLA class I genes, have been associated with outcome in many human infections, notably Plasmodium falciparum, Mycobacterium tuberculosis, HIV-1, HTLV-1 and Hepatitis B Virus infection. The benefit of a genotypic analysis is that the direction of causality is unequivocal; the drawback is that, in common with all “omics” approaches to identify biomarkers, mechanistic insight is limited. Provided linkage disequilibrium can be ruled out, class I associations imply that the protective effect is mediated by CD8^+^ T or NK cells. However, why one particular allele should be protective remains unclear and so a class I association provides no information about how to manipulate the immune response to enhance protection.

The aim of this study was to develop a method to test the hypothesis that the effectiveness of an individual's HTLV-1-specific response and thus their proviral load and HAM/TSP risk was determined by the epitope binding properties of their HLA class I alleles. This method resulted in the identification of the viral protein HTLV-1 basic leucine zipper factor (HBZ) as a significant immunogenic target for both proviral load reduction and reduced disease risk. The HBZ gene was identified recently [20], it is encoded by the complementary strand of the HTLV-1 genome and its promoter lies in the 3′ LTR rather than the 5′ LTR. Our approach is generally applicable to all pathogens, including those in which few epitopes have been identified experimentally.

## Results

### Verification of epitope prediction software

**Figure 1:**
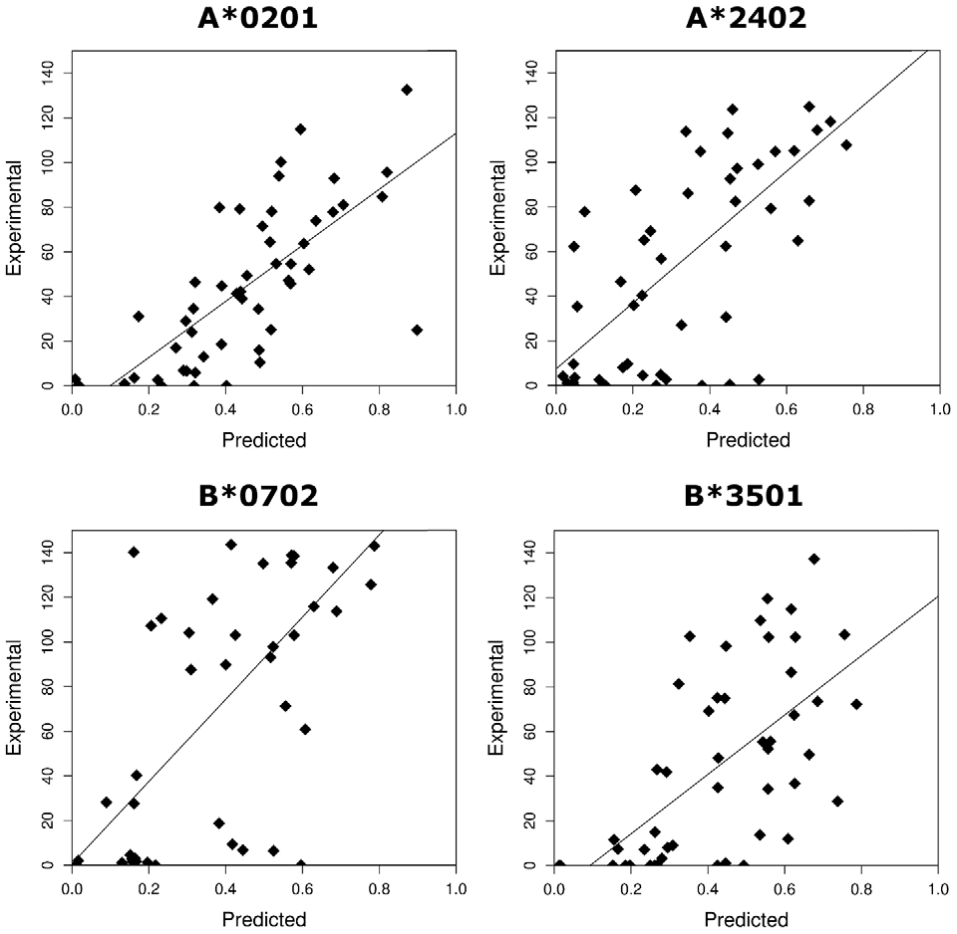
The correlation between the experimentally measured binding affinities (% binding compared to control peptide) and the predicted binding affinities (1-log (affinity)) from Metaserver of 200 HLTV-1 peptides to 4 HLA class I molecules. A*0201: R_S_ = 0.76, *P* = 1×10^−10^; B*0702: R_S_ = 0.62, *P* = 9×10^−6^; A*2402: R_S_ = 0.65, *P* = 5×10^−7^; B*3501: R_S_ = 0.68, *P* = 9×10^−8^.

Approximately 50 HLA class I-epitope pairs have been identified for HTLV-1 [12], [21]–[23] (mainly from the immunodominant protein Tax [24] in the context of A*02); this represents a small and non-random fraction of the ∼2200 nonamer epitopes that could be bound by the alleles of the Kagoshima cohort studied here (Methods). Therefore we used epitope prediction software to systematically predict HTLV-1 epitopes. The epitope prediction software that we used has been extensively validated for a number of other organisms including HIV-1 where it has provided useful insight [25]–[31], but because of the lack of experimental data, it has not previously been tested for HTLV-1. To validate the epitope prediction software, we measured experimentally the binding affinity of 200 HTLV-1 peptide-allele combinations (Table S1 in Supporting Information S1). We found a strong positive correlation between experimental measurement and the theoretical prediction for each of the two epitope prediction methods used namely Metaserver and Epipred (Metaserver: all *P* <0.00001, Spearman's rank correlation; Fig. 1. Epipred: all *P* <0.001, Spearman's rank correlation; Fig. S1 in Supporting Information S1). We conclude that these epitope prediction software packages accurately predict relative (i.e. rank order) HTLV-1 peptide binding affinities. Throughout this article figures in the main text are obtained using Metaserver, corresponding figures from Epipred are in Supporting Information S1. All conclusions were replicated by both methods and by an alternative metric (Supporting Information S1).

### Protective class I alleles bind HBZ strongly

**Figure 2:**
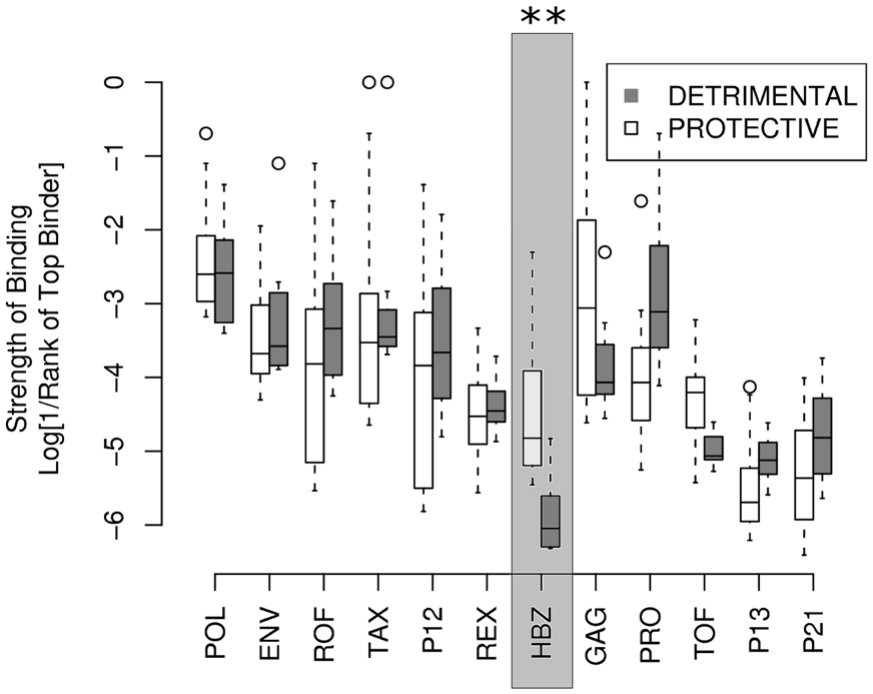
The strength of binding of protective alleles (A*0201 and Cw*0801) and detrimental allele (B*5401) across the 12 HTLV-1 proteins. The y-axis gives strength of binding of the top 8 binding peptides from each protein to each of the alleles. The level of significance indicated is corrected for multiple comparisons and provided in Table S2 in Supporting Information S1. Repeating the test with the top 5 or the top 10 instead of the top 8 peptides yielded identical conclusions.

A number of associations between HLA class I alleles and proviral load or HAM/TSP risk in HTLV-1 infection have been identified in a population in southern Japan [3], [32]. We compared the predicted HTLV-1 peptide-binding affinities of the two protective alleles, A*0201 and Cw*0801, with those of the known detrimental allele, B*5401 (Methods). Peptides from the HTLV-1 protein HBZ bound to HLA-A*0201 and Cw*0801 significantly more strongly compared to B*5401 (*P* = 0.0002, Wilcoxon-Mann-Whitney; Fig. 2. Repeating the analysis with another protective allele from the A*02 family, namely A*0206 instead of A*0201 yielded identical conclusions *P* = 0.0007, Wilcoxon-Mann-Whitney, data not shown). These *P* values needs to be treated with caution because the rank of the binding affinity of one HBZ peptide for A*0201 may not be independent of the rank of the binding affinity of a second peptide to A*0201 and similarly for Cw*0801 and B*5401 (see Methods, independence of ranks). However, we also found that the difference in binding strength (i.e. the rank of the top A*0201 binding peptide minus the rank of the top B*5401 binding peptide) was significantly greater for HBZ than for other HTLV-1 proteins (*P* <0.001, binomial test). This statistic is based only on the top binding peptide so it does not assume different peptides have independent binding affinity ranks. Henceforth, we only considered the top binding peptide to avoid the potential problem of dependence (Methods).

### Asymptomatic carriers bind HBZ more strongly than HAM/TSP patients

**Figure 3:**
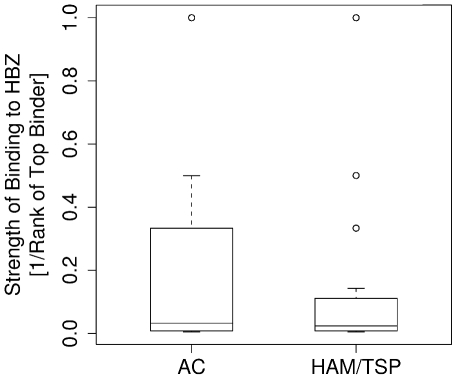
The strength of binding of the HLA class I alleles of asymptomatic carriers and HAM/TSP patients to HBZ. Asymptomatic carriers have HLA class I alleles that are predicted to bind HBZ significantly more strongly than HAM/TSP patients (*P* = 0.0002).

Having established that the known protective HLA class I alleles code for molecules that bind to peptides from HBZ more strongly than the known detrimental allele, we examined peptide binding by all alleles in the Kagoshima cohort. We compared the predicted epitopes for asymptomatic carriers (*n* = 202) and HAM/TSP patients (*n* = 230) from the Kagoshima cohort. We predicted the HTLV-1 peptides bound most strongly by each individual, given their HLA class I types and then tested for differences between the two subject groups (Methods). The results are shown in Table S2 in Supporting Information S1. One result remained highly statistically significant after correction for multiple comparisons and was consistent across both prediction methods: asymptomatic carriers have HLA class I alleles that bind more strongly to peptides from HBZ compared to HAM/TSP patients (Metaserver: *P* = 0.0002, Wilcoxon-Mann-Whitney; Fig. 3. Epipred: *P* <0.0001, Wilcoxon-Mann-Whitney; Fig. S2 in Supporting Information S1).

To test whether this association was caused solely by the known protective and detrimental HLA allele families, the analysis for HBZ was repeated excluding A*02 and B*54. The results showed that, amongst the HLA-A alleles, alleles from the A*02 family were responsible for the protective effect, whereas in HLA-B more than one allele family contributed significant effects. Overall, strong binding of HBZ peptides was associated with asymptomatic status, even when A*02, B*54 and Cw*08 were excluded from the analysis (Metaserver: *P* = 0.04, Wilcoxon-Mann-Whitney. Epipred: *P* = 0.006, Wilcoxon-Mann-Whitney; Table 1).

**Table 1:** The difference in binding strength to HBZ between HAM/TSP patients and asymptomatic carriers.

		Whole cohort (N = 202, 230)	Excluding A*02 & B*54 (N = 84,116)
Metaserver	A alleles	0.006	0.81
	B alleles	0.001	0.01
	Combined	**0.0005**	**0.04**
Epipred	A alleles	0.0009	0.72
	B alleles	0.0002	0.001
	Combined	**0.000001**	**0.006**

The first column gives the *P* values of the Wilcoxon-Mann-Whitney tests for the A and B loci. The second column repeats this analysis excluding individuals with alleles from either the A*02 or B*54 allele families.

### Individuals whose HLA class I genotype predisposed them to bind HBZ peptides strongly had a significantly lower proviral load

**Figure 4:**
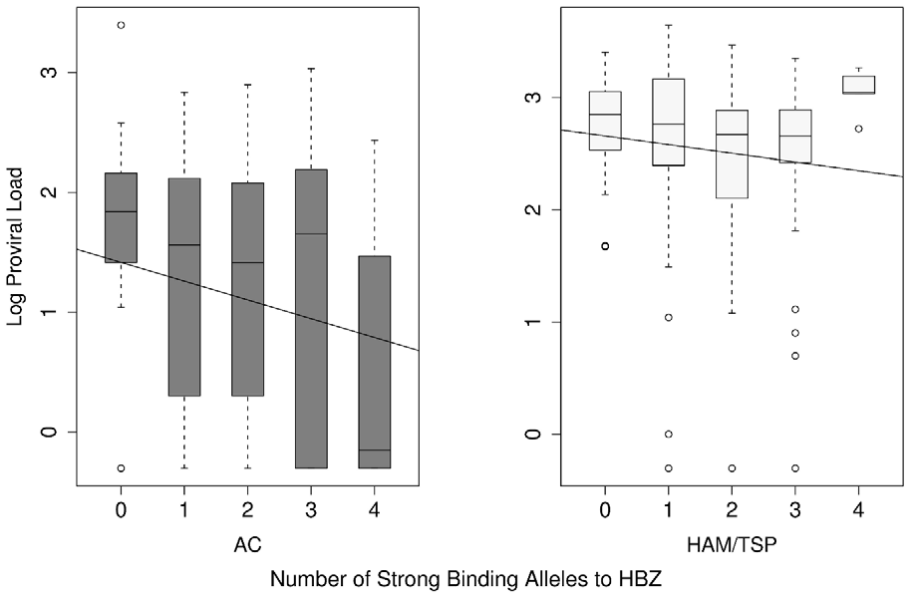
The count of strong binding alleles to HBZ per individual against their proviral load in AC and HAM/TSP groups. The number of HLA class I alleles that are strong binders to HBZ is significantly negatively correlated with proviral load (*P* = 0.016). This analysis was performed separately in HAM/TSP patients and ACs so it does not follow trivially from our previous observation that ACs (who have lower proviral load than HAM/TSP patients) show stronger binding to HBZ.

Next we investigated why strong binding of HBZ peptides was associated with remaining asymptomatic. One of the strongest correlates of HAM/TSP is a high HTLV-1 proviral load [33]. We therefore tested the hypothesis that strong binding of HBZ peptides was associated with a lower proviral load. The number of HLA class I alleles that each individual possessed that were predicted to strongly bind peptides from HBZ was plotted against their proviral load (Methods). We found that the number of HLA Class I alleles that an individual had that strongly bound HBZ peptides was significantly negatively correlated with their proviral load (Metaserver: *P* = 0.016, Spearman's rank correlation; Fig. 4. Epipred: *P* = 0.1, Spearman's rank correlation; Fig. S3 in Supporting Information S1). We tested this correlation independently in HAM/TSP patients and asymptomatic carriers and then combined the *P* values (rather than simply testing the whole cohort), so this result does not follow trivially from our previous observation than asymptomatic carriers bind HBZ significantly more strongly than HAM/TSP patients. An alternative metric, the binding strength of the top HBZ-binding peptide to each allele instead of the number of strongly binding alleles, yielded an identical conclusion i.e. there was a significant negative correlation between the proviral load and the strength of binding to HBZ peptides (Metaserver: *P* = 0.008, Spearman's rank correlation. Epipred: *P* = 0.003, Spearman's rank correlation).

### HBZ peptide binding is a consistent predictor of proviral load

Next we compared our peptide-binding analysis of HLA class I genotype with a traditional frequency-based “presence or absence of an allele” analysis. Previously a “traditional” analysis yielded inconsistent results [3], [32], [34]. For example, A*02 was a significant predictor of load in ACs but not in patients with HAM/TSP. We therefore directly compared the ability of the novel peptide binding method and the traditional genotype method to predict proviral load in ACs and HAM/TSP patients (Table S3 in Supporting Information S1). This analysis showed that binding HBZ was a significant predictor of proviral load in both ACs and HAM/TSP patients (*P* = 0.001, *P* = 0.017), but confirmed the finding that in a traditional analysis HLA-A*02 (presence/absence) was a significant predictor in ACs only (*P* = 0.01) and HLA-B*54 for HAM/TSP patients only (*P* = 0.019). The proportion of variance in proviral load explained was also marginally higher for the peptide binding analysis than the traditional analysis. The observation that HBZ binding strength correlated with proviral load in both ACs and HAM/TSP patients suggests that peptide binding is the more fundamental predictor than HLA genotype.

### HLA class I binding of peptides from different HTLV-1 proteins has a differential and correlated impact on both proviral load and HAM/TSP risk

**Figure 5:**
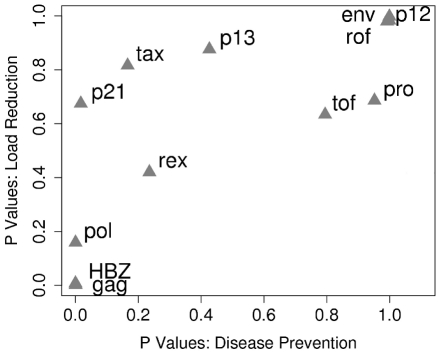
HLA class I binding of peptides from different HTLV-1 proteins has a differential and correlated impact on both proviral load and HAM/TSP risk. The HTLV-1 proteins were ranked according to whether they were bound significantly more strongly by asymptomatic carriers or HAM/TSP patients (x-axis; at the extremes ACs were significantly more likely to bind peptides from HBZ, HAM/TSP patients were significantly more likely to bind peptides from Env). This list could be viewed as the “rank order of targets for a vaccine designed to reduce HAM/TSP risk”. Proteins were also ranked according to whether binding their peptides was associated with a lower proviral load (y-axis; at the extremes binding of HBZ was associated with a significantly lower proviral load, binding of Env was associated with a significantly higher proviral load). This list could be viewed as the “rank order of targets for a vaccine designed to reduce proviral load”. These two sets of ranks were positively correlated (R_S_ = 0.86, P = 0.0005, Spearman's rank correlation). That is, proteins whose peptides are bound by asymptomatic carriers (left hand side of the graph) are, independently, those associated with a lower proviral load when bound (bottom of the graph).

Our findings demonstrate that the HTLV-1 protein that is associated with the most significant reduction in HAM/TSP risk when bound by HLA class I molecules (i.e. HBZ) is also, independently, associated with a significant reduction in proviral load when bound. We wished to investigate whether this relationship held across all proteins. We therefore produced two ranked lists of proteins. In the first list we ranked the HTLV-1 proteins according to whether they were bound more strongly by asymptomatic carriers or HAM/TSP patients (Fig. 5 x-axis; at the extremes ACs were significantly more likely to bind peptides from HBZ, HAM/TSP patients were significantly more likely to bind peptides from Env). This list could be viewed as the rank order of targets for a vaccine designed to reduce HAM/TSP risk. In the second list we ranked the proteins according to whether binding their peptides was associated with a lower proviral load (Fig. 5, y-axis; at the extremes, binding of HBZ was associated with a significantly lower proviral load, whereas binding of Env was associated with a significantly higher proviral load). This list could be viewed as the rank order of targets for a vaccine designed to reduce proviral load. We then compared these two sets of ranks and found them to be strongly positively correlated (Metaserver: R_S_ = 0.86, P = 0.0005, Spearman's rank correlation; Fig. 5. Epipred: R_S_ = 0.66, P = 0.02, Spearman's rank correlation; Fig. S4 in Supporting Information S1). That is, proteins whose peptides are bound strongly by asymptomatic carriers are, independently, those associated with a lower proviral load when bound. This observation has two important implications. Firstly, HLA class I binding of peptides from different proteins has a differential impact on both proviral load and HAM/TSP risk; i.e. CD8^+^ efficiency (ability to reduce proviral load and disease risk) is determined by protein specificity and we have established a list of protein targets of the most efficient response to the least efficient response. Secondly, the fact that across all alleles and across all proteins, peptide binding associated with immune control (reduced proviral load) is strongly correlated with prevention of HAM/TSP is the strongest evidence yet that the CD8^+^ T cell response can have a beneficial role in HTLV-1 infection.

### The prevented fraction of disease, F_P_

We calculated the prevented fraction of disease attributable to the possession of one or more strong binding alleles to HBZ [3] (Methods). This showed that the possession of strong HBZ-binding HLA alleles prevented (F_p_)≈48% (12.3% SD) of potential cases of HAM/TSP in the study population. However, although we found that a high proportion of potential HAM/TSP cases are prevented by strong HBZ binding, it should be noted that the strength of HBZ binding is not the only determinant of disease status: in a logistic regression model, the strength of HBZ binding alone could only correctly classify 55% of cases of HAM/TSP.

### HBZ-specific CD8^+^ T cells can be detected *ex vivo*

**Figure 6:**
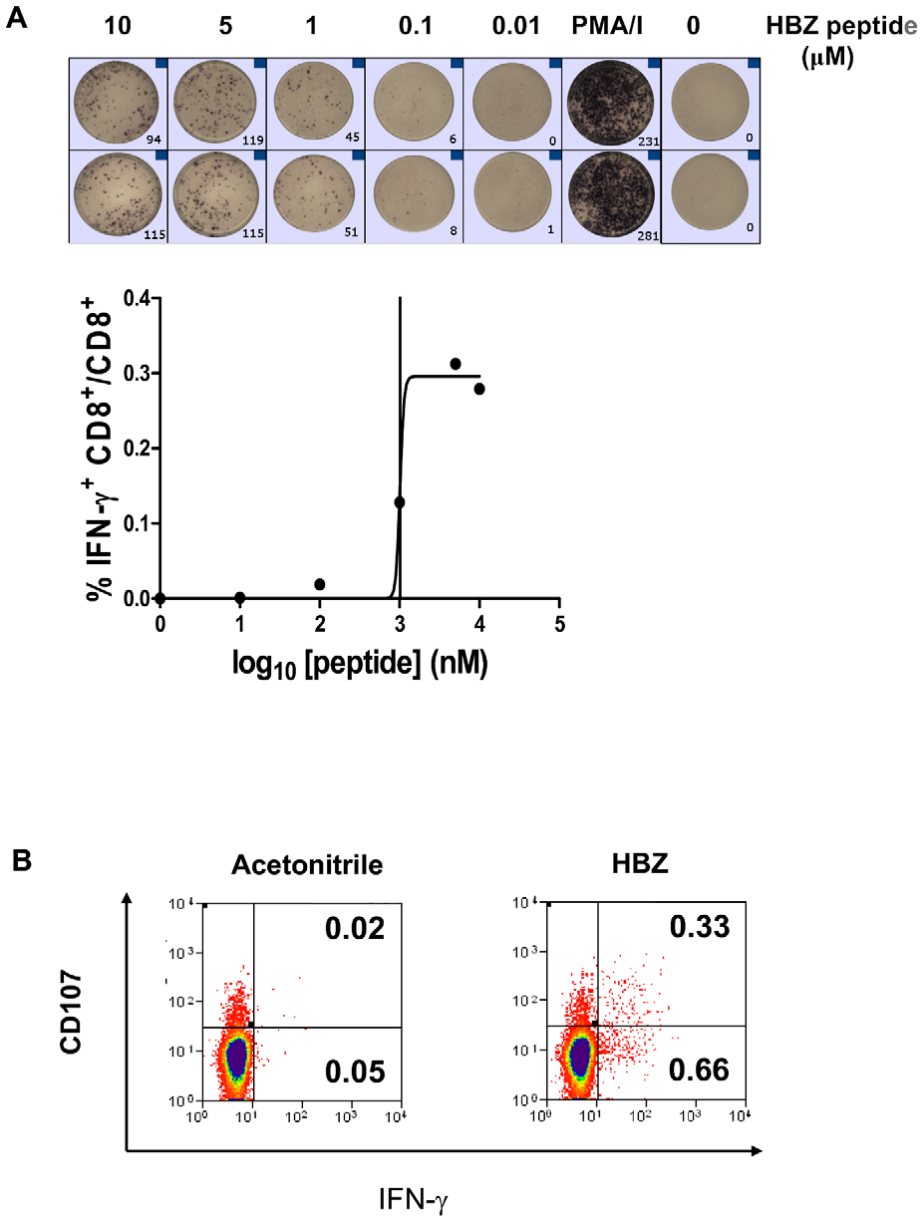
HBZ-specific CD8^+^ T cells are directly detectable ex vivo. (**A**) *CD4-depleted PBMC from ACs and HAM/TSP patients produce IFNγ in response to HBZ peptide*. CD4-depleted PBMC were cultured in the presence of HBZ peptide. After 6hrs IFNγ producing cells were detected by ELISpot. The threshold for a positive response to peptide was defined as greater than the mean plus two standard deviations of the number of spots in the medium only control. (**B**) *CD8^+^ T cells from ACs and HAM/TSP patients degranulate in response to HBZ*. PBMC were cultured in the presence of anti-CD107a and monensin, in the presence of 2 µM HBZ peptide or its solvent, acetonitrile. After 5h, cells were harvested and stained. Dot plots shown are gated on the CD3^+^CD8^+^ live population.

These results strongly imply that HBZ-specific CD8^+^ T cells play a protective role in HTLV-1 infection. HBZ immunogenicity has been studied in ATL patients [35], [36] but it is unknown whether a HBZ-specific CD8^+^ T cell response is generated or even whether HBZ protein is expressed in asymptomatic carriers and HAM/TSP patients. We therefore sought to identify HBZ-specific CD8^+^ T cells in fresh PBMCs from HTLV-1 infected individuals. We assayed IFN-γ production by ELISpot following stimulation *in vitro* with a pool of overlapping peptides that spanned the entire HBZ protein. Of 45 subjects tested, 31% had detectable HBZ-specific CD8^+^ T cells (Fig. 6). An independent CD8^+^ T cell assay, (CD107a mobilisation), confirmed that HBZ-specific CD8^+^ T cells are present in PBMC from infected individuals. We conclude that HBZ protein is expressed *in vivo* and is immunogenic.

### Naturally infected cells can be lysed by an HBZ-specific CD8^+^ T cell clone

**Figure 7:**
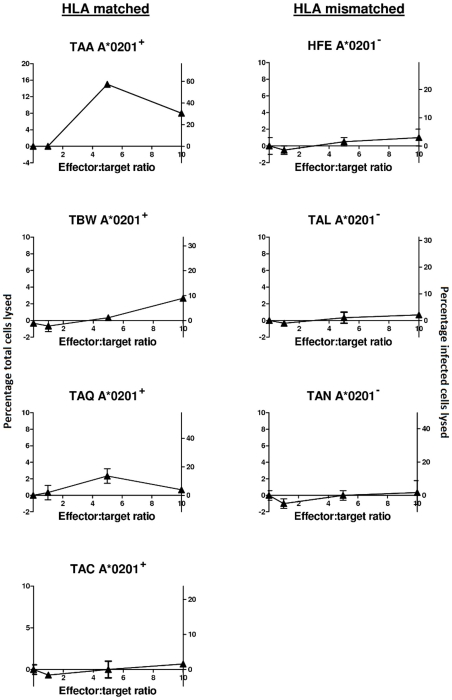
Naturally infected cells from ACs and HAM/TSP patients are susceptible to lysis by an HBZ-specific A*0201-restricted clone. Target CD4^+^CD25^+^ cells from four HLA-A*0201^+^ and three HLA-A*0201^−^ HTLV-I infected individuals were labelled with ^51^Chromium and mixed with an HBZ-specific clone at the indicated effector∶target ratios in triplicate. The cells were co-cultured for 4h, after which target lysis was detected by chromium release into the supernatant. Only a small proportion of CD4^+^CD25^+^ cells will be infected so as well as expressing lysis as percentage of total CD4^+^CD25^+^ cells lysed (primary, left hand y axis) we also estimated the proportion of provirus-positive cells lysed (secondary, right hand y axis). We found that naturally infected targets were susceptible to lysis in 3 out of 4 HLA matched individuals (first column) but in 0 out of 3 HLA-mismatched individuals (second column). Maximum lysis in the 3 responding individuals was in the range 10–30% of provirus-positive cells lysed in 4h. Proviral load in the 7 individuals is as follows: TAA 3.5, TBW 5.67, TAQ 3, TAC 10, HFE 6.27, TAL 9.1, TAN 4 copies per 100 PBMC.

Recently, Sumeori et al established an HBZ-specific CD8^+^ T cell clone that recognised HBZ_26–34_ (GLLSLEEEL) in the context of HLA-A*0201 [37]. They showed that this clone was able to lyse an autologous B-lymphoblastoid cell line (B-LCL) that had been loaded with HBZ peptide but that cells from an ATL patient were resistant to killing. We investigated whether the same CD8^+^ T cell clone was able to kill naturally-infected cells from non-leukemic HTLV-I-infected individuals. First we confirmed the finding of Sumeori et al that autologous B-LCL loaded with HBZ_26–34_ peptide could be lysed by the CD8^+^ T cell clone (data not shown). Then we demonstrated, by a classical chromium release assay, that naturally-infected CD4^+^CD25^+^ cells from the PBMCs of 3 out of 4 HLA-A*0201^+^ non-leukemic patients were lysed by the CD8^+^ T cell clone but that cells from 3 out of 3 HLA-mismatched donors were not lysed (Fig. 7). We conclude that naturally-infected cells from AC and HAM/TSP patients are susceptible to lysis by an HBZ-specific clone.

### The comparative immunogenicity of HBZ and Tax

**Figure 8:**
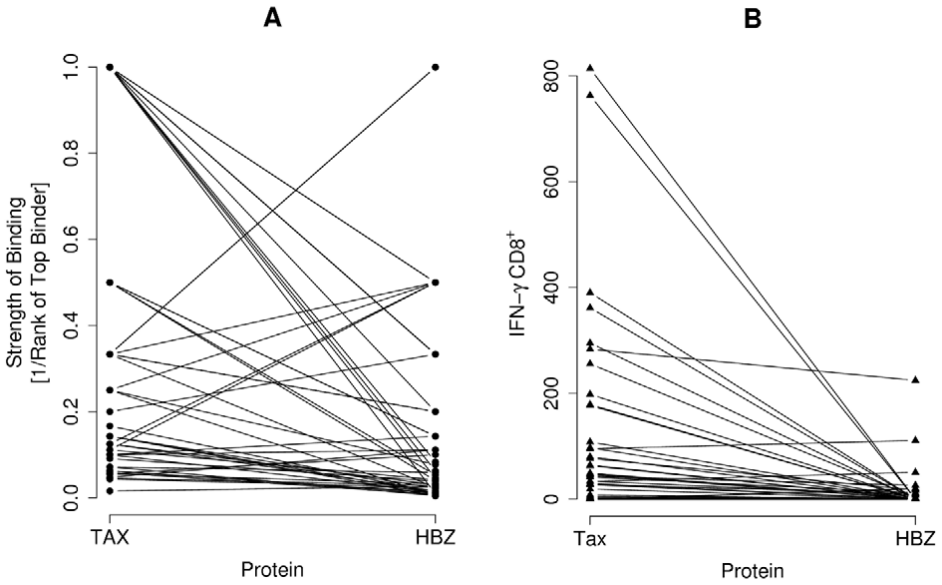
The comparative immunogenicity of HBZ and Tax. (**A**) The predicted top binding peptide from Tax and HBZ to each of the 43 alleles for which Metaserver predicts binding affinities was found. Peptides from Tax are bound more strongly than peptides from HBZ (*P* = 0.00002, paired Wilcoxon-Mann-Whitney). Data pairs represent binding of the same allele to the two different proteins Tax and HBZ. (**B**) Consistent with this, the frequency of Tax-specific CD8^+^ T cells was also greater compared to HBZ-specific CD8^+^ T cells in the 45 HTLV-1 infected individuals tested using IFN-γ ELISpot (*P* = 0.000006, paired Wilcoxon-Mann-Whitney). Data pairs represent frequency of the response in the same individual to the two different proteins Tax and HBZ.

How does the immunogenicity of HBZ compare to Tax? We compared the predicted top binding peptide from HBZ and Tax respectively to 43 HLA class I alleles (the maximum capacity of Metaserver). Peptides from Tax were predicted to bind significantly more strongly than peptides from HBZ (*P* = 0.00002, paired Wilcoxon-Mann-Whitney; Fig. 8A). Consistent with this prediction, the frequency of Tax-specific CD8^+^ T cells by IFN-γ ELISpot was also significantly greater compared to HBZ CD8^+^ T cells in 45 HTLV-1-infected individuals (*P* = 0.000006, paired Wilcoxon-Mann-Whitney; Fig. 8B).

## Discussion

We show that strong predicted binding of peptides from the HTLV-1 protein HBZ is associated with a reduced risk of HAM/TSP and a reduced proviral load in a population with endemic HTLV-1 infection in southern Japan. We demonstrated that protection is not limited to a small subset of HLA class I alleles previously associated with disease status and proviral load (HLA-A*02 and HLA-Cw*08), but is generally associated with HLA class I alleles that bind strongly to HBZ. Given that a protein-specific HLA-restricted association is more likely to be mediated by CD8^+^ T cells than NK cells which show limited protein specificity we interpret this work in the context of the CD8^+^ T cell response.

Prior to this analysis, CD8^+^ T cells specific for the HTLV-1 protein Tax were often considered as the best candidate for ‘efficient’ or ‘protective’ CD8^+^ cells because of the immunodominance of Tax in the CD8^+^ T cell response [9], [24]. Our finding that binding of HBZ peptides rather than Tax peptides is protective raises the question: why is HBZ a critical target for the immune response?

HBZ functions by binding to cellular factors of the JUN and ATF/CREB families [37]. There are two major splice variants of the HBZ transcript, SP1 and SP2; the variant SP1 is more abundant and is the variant used in this study [38], [39]. The abundance of HBZ transcript has been previously correlated with disease severity [39]. Expression of HBZ suppresses Tax-mediated transactivation through the 5′ LTR [20], [40] and thereby inhibits expression of other HTLV-1 genes [20], [41]; HBZ can be expressed in the absence of transcription of other HTLV-1 genes. Additionally, HBZ RNA promotes the proliferation of infected T-lymphocytes [35]. This dual action – reduction of HTLV-1 expression and subsequent protection from immune surveillance, and enhancement of infected cell proliferation – probably confers a survival advantage on HBZ-expressing cells and is consistent with the observations that HBZ enhances persistence in HTLV-1 inoculated rabbits [41] and that ATL cells often have a hypermethylated or deleted 5′ LTR but an intact functional 3′ LTR [35]. We hypothesise that if HBZ-specific CD8^+^ T cells are weak or absent then infected cells that express HBZ but not other viral proteins will evade immune surveillance and proliferate rapidly, leading to an increase in proviral load. HBZ-specific CD8^+^ T cells would then play an important role in preventing this proliferation of provirus-positive cells and blocking this strategy of persistence. If this conclusion is correct that CD8^+^ T cell recognition of HBZ plays a central role in the control of HTLV-1 replication then one might expect that HBZ would have evolved to minimize class I binding. Consistent with this hypothesis, we find that the predicted binding affinity of HLA molecules to HBZ peptides is significantly weaker than that of Tax peptides and that the frequency of HBZ-specific CD8^+^ T cells is significantly lower than the frequency of Tax-specific CD8^+^ T cells. Although the low immunogenicity of HBZ is precisely what we predict given its central importance in maintaining HTLV-1 persistence, it is nevertheless striking that these low T cell frequency responses are so important. This result challenges the prevailing assumption that the immunodominant response to a pathogen is the most important.

We demonstrated using two different assays (IFNγ ELISpot and CD107 mobilisation) that HBZ-specific CTL are present in PBMC from HAM/TSP patients and ACs. We further show that naturally infected cells, isolated directly from HAM/TSP patients and ACs, are susceptible to lysis by an HBZ-specific CTL clone. Suemori *et al* have previously reported that the same HBZ-specific CTL clone was unable to lyse leukemic cells isolated from a patient with adult T cell leukemia [37]. The observation that aleukemic but not leukemic cells can be lysed may be because leukemic cells express lower levels of HLA: HBZ peptide on their surface or because leukemic cells can be inherently harder to lyse [42]–[44].

This approach to studying the association between HLA class I genotype and the outcome of infection has a number of strengths compared with a traditional frequency-based analysis. Firstly, it is more mechanistic: knowing that binding HBZ is associated with a reduced proviral load and disease risk compared with knowing that A*02 is associated with these outcomes is a simultaneously more fundamental and more applicable level of understanding. Secondly, identification of protective epitopes immediately suggests a practical approach to measure and enhance, via therapeutic vaccination, the efficiency of an individual's anti-viral response. Thirdly, because the same effect (e.g. HBZ binding) can be identified for many alleles it is less likely to be a spurious result of linkage disequilibrium or genetic stratification. Finally, effects due to multiple low-frequency alleles can be captured because analysis is made at the level of peptide binding rather than allelic frequency.

In summary, using a novel and generalizable approach, we have identified one of the constituents of an effective CD8^+^ T cell response in HTLV-1 infection.

## Methods

### Subjects

#### Kagoshima cohort

Two hundred and thirty individuals with HAM/TSP were compared with two hundred and two randomly selected HTLV-1 seropositive asymptomatic carriers (ACs) from the Kagoshima Red Cross Blood Transfusion Service. All cases and controls were of Japanese ethnic origin and resided in Kagoshima Prefecture, Japan. Full details of the cohort can be found in [3]. Each individual was HLA class I typed using PCR–sequence-specific primer reactions. Their proviral load in peripheral blood mononuclear cells was measured by quantitative PCR.

#### St Mary's cohort

IFNγ ELISpot, CD107a mobilisation and the chromium release assays were all carried out using cells from HTLV-1-infected individuals attending St Mary's Hospital. **Ethics statement:** All subjects attended the National Centre for Human Retrovirology at St. Mary's Hospital and donated blood after giving written informed consent as approved by the St. Mary's Hospital Research Ethics Committee.

### Epitope prediction

We used two different algorithms to predict HLA class I epitopes: Metaserver and Epipred. Figures based on Metaserver predictions are in the main text, the corresponding figures for Epipred are in Supporting Information S1.

#### Metaserver

Metaserver is a combination of two web-based prediction methods that use artificial neural nets, NetCTL v1.2 [45] and NetMHC v3.0 [46], [47]. NetCTL is an integrated method that predicts TAP transport, proteasomal cleavage and HLA binding for 12 different class I alleles. NetMHC v3.0 predicts HLA-peptide binding for 43 HLA molecules. Metaserver combines the two methods and removes a normalising assumption (which held that all alleles bind the same number of peptides) to produce a technique that shows improved accuracy in epitope prediction [48] and predicts epitopes for 43 HLA molecules.

#### Epipred

In order to validate our results, we used a second, independent method of epitope prediction [49]. Epipred uses a logistic regression model that is trained on all available data across all HLA class I alleles and then specified for an individual allele.

### Epitope prediction - allele coverage

Other than our initial comparison (protective against detrimental alleles), analysis was limited to A and B loci for two reasons: Metaserver does not have algorithms for the C loci and C loci predictors tend to be less accurate because of the lack of peptide-HLA-C experimental binding affinities to train the software. Metaserver provided coverage of 84% of the total count of A/B alleles in the Kagoshima cohort.

The missing alleles were: A*0207, A*0210, A*2603, A*3201, B*1301, B*1501, B*1508, B*1511, B*1518, B*2704, B*3701, B*3802, B*4005, B*4006, B*4601, B*4801, B*5201, B*5501, B*5504, B*5601, B*5603, B*5605, B*5705, B*5901, and B*6701.

We were able to obtain predictions for [A*0207, A*0210], A*2603 and [B*4005, B*4006] to a resolution of 2 digits by combining the predictions of other A02*, A26* and B40* predictors according to their frequency in Kagoshima.

### Estimated number of epitope-allele combinations

We estimate that approximately 2,200 peptides could be bound by the alleles present in the Kagoshima cohort. This figure is 1% [45] of the 3,389 overlapping nonamers of the HTLV-1 proteome multiplied by the number of unique alleles (65) in the cohort.

### Prediction quality

The accuracy of epitope prediction algorithms has increased to such an extent that the correlation between predicted binding affinities and measured binding affinity is as strong as the correlations of measurements between different laboratories [50]. The specificity of epitope predictors has been tested by predicting a set of CTL epitopes and subsequently verifying CD8^+^ T cell responses against these epitopes experimentally. Using this technique has yielded true-positive (correctly predicted) estimates of 62–80% [51]. Using the more direct approach of mass spectrometry to determine HLA-peptide binding yielded a true positive rate of greater than 98% [52]. Additionally, we verified the prediction software we used (Metaserver and Epipred) for HTLV-1 peptides.

### The rank measure

Both prediction methods that we use produce a score for each peptide-HLA that represents the binding strength of that complex. In theory this score would allow us to compare predicted binding affinities between alleles. However, between allele comparisons can be problematic. Firstly, within-allele comparisons (i.e. predictions for different peptides to the same allele) are thought to be more comparable than predictions between alleles [45]. Secondly, whether or not a normalisation procedure should be applied for between-allele comparisons is still being debated in the community [48]. To avoid the potential problem of between-allele comparisons we used the rank measure technique introduced by Borghans et al. [53] in which she quantified the strength of peptide-HLA class I binding for peptides from a particular protein by ranking the binding score of peptides from the protein of interest to the allele amongst the binding score of peptides from the entire proteome to that allele; this approach has been successfully applied in the context of HIV infection [25], [28]. Specifically, we split each protein in the HTLV-1 reference sequence into overlapping nonamers offset by a single amino acid. Using the epitope prediction software, a predicted binding affinity score was calculated for each of these peptides to each HLA allele of interest. For each allele we ranked all nonamers from the proteome from the strongest to weakest predicted binding scores. This produced a list of rank values for each protein to that particular allele that quantified the binding relationship between that allele and the protein (an example is given in Table S4 in Supporting Information S1). To check for robustness we also repeated all calculations using an alternative to the rank measure: the raw predicted affinity score. We found that our conclusions were robust to the choice of method (Table S5 in Supporting Information S1).

### Independence of ranks

We were concerned that the binding of the top 8 peptides from a protein to an allele may not be independent of one another. Since, the strength of the strongest binder provides information (i.e. an upper bound) about the strength of the second highest binder. For this reason, apart from Fig. 2, only the top rank for each protein-allele pair was used.

### Experimental quantification of HLA class 1-peptide binding

The REVEAL HLA-peptide binding assay (ProImmune Ltd., Oxford, UK) was used to quantify peptide-HLA binding. For each allele-peptide combination that was tested, assembly of peptide-HLA complexes was quantified by ELISA with a conformation-dependent anti-HLA antibody. Samples of assembling peptide-HLA complexes were taken at a defined time point and snap-frozen in liquid nitrogen prior to analysis. The assembly for each peptide-HLA complex was then compared against a positive control peptide for that allele as the percentage of assembled peptide relative to that control. We selected four HLA class I alleles and 50 HTLV-1 peptides for each allele. The allele choice was based on allele frequency in the Kagoshima database and included 2 A alleles and 2 B alleles as well as alleles for which we knew that the epitope prediction tended to be poor. The 50 HTLV-1 nonamer peptides for each allele were selected to represent a range of predicted binding affinities, from weak to strong binding peptides. They originated from 4 HTLV-1 reference strain proteins: Tax, HBZ, Gag and Polymerase.

### Protective versus detrimental alleles

Due to allele coverage (see above), it was necessary to use Metaserver for A*0201 and B*5401 and Epipred for Cw*0801. As the rank values were derived for each allele separately, it was acceptable to use different prediction methods for each allele in this case. Epipred predicts binding to allele families rather than individual alleles and so we calculated binding to Cw*08. The ranks of the strongest binding 8 peptides from each protein to the alleles A*0201 and Cw*08 (16 rank values) were compared against the ranks of the strongest binding 8 peptides to the allele B*5401 (8 rank values). A Wilcoxon-Mann-Whitney test was performed for each protein to test for differences between the two sets of rank values. The analysis was repeated using top 5 and top 10 as well as top 8 binding peptides, conclusions were robust to the choice of number of peptides (Results in Supporting Information S1). Finally, to avoid the potential problem of lack of independence of ranks (see “independence of ranks” above) we performed a binomial test on the difference in strength of binding of A*02 and B*54 to HBZ compared to all other HTLV-I proteins. The null hypothesis we tested was “the difference in binding of detrimental and protective alleles to HBZ is comparable to the other HTLV-1 proteins”. For each of the 12 HTLV-1 proteins we calculated the ranks of the single highest ranking peptide from that protein to A*02 and B*54. We then calculated the difference of these two ranks (detrimental – beneficial) for each of the 12 proteins and asked, using the Binomial test, whether the difference in binding for HBZ was larger than would be expected under the null hypothesis.

### HAM/TSP versus asymptomatic carriers

The analysis was carried out on each HTLV-1 protein in turn. For each individual in the Kagoshima cohort, the rank of the top binding peptide from the HTLV-1 protein to each of the individual's A and B HLA class I alleles was found (see The Rank Measure). These ranks were then split into two groups – those from HAM/TSP patients and those from asymptomatic carriers (AC). The two sets of ranks (HAM/TSP vs. AC) were then compared for each protein using a Wilcoxon-Mann-Whitney test (null hypothesis: HAM/TSP patients and asymptomatic carriers bind the protein equally strongly).

### Rank versus proviral load

We considered each HTLV-1 protein in turn. Firstly, we split the cohort by disease status (AC or HAM/TSP). Then, for each individual, we counted the number of alleles they possessed that were strong binders to the protein of interest and then tested for a correlation between the number of strong binders to the protein and proviral load using the Spearman rank correlation. A strong binding allele to a particular protein was defined as one that was in the top 40% of alleles. That is, the rank of the top binding peptide from the HTLV-1 protein to each of the individual's A and B HLA class I alleles was found (see The Rank Measure). This set of rank values (pooled HAM/TSP and AC) was then ordered from highest to lowest rank and the alleles that were represented in the top 40% of these ranks were defined as strong binding alleles to that protein. Importantly, for each protein, we looked at the relationship between strength of binding and proviral load separately in HAM/TSP patients and ACs and then combined the *P* values using Fisher's combined test (rather than simply looking at the relationship in the whole cohort). Therefore we could be confident that any relationship between protein binding and proviral load that we found did not follow trivially from a relationship between protein binding and disease status and the fact that asymptomatic carriers have a significantly lower load than HAM/TSP patients.

Our alternative metric for this method used the Rank Measure to quantify the strength of binding of peptides from each HTLV-1 protein to each individual's A and B alleles. We then tested for any correlation between these values and the individuals' proviral load for HAM/TSP patients and asymptomatic carriers.

### Robustness of conclusions

All analysis was performed with two independent epitope prediction algorithms (Metaserver and Epipred) and with two different methods (rank method, raw score method); additionally an alternative approach to comparing protective v detrimental alleles (based on the binomial test) and to comparing proviral load with strength of binding were investigated. Conclusions were highly robust (Table S5 in Supporting Information S1).

### Statistical analysis

All statistical analysis was carried out using the R Project for Statistical Computing [54]. The tests were non-parametric with the exception of multiple linear regression. All *P* values reported are 2-tailed. Fisher's combined probability test was used to combine *P* values.

### Multivariate regression

General linear model analysis [55] was used to identify which factors were predictors of proviral load, either in ACs or patients with HAM/TSP.

### Prevented fraction of disease, F_P_

To calculate the prevented fraction (F_p_) of disease [3], [56], we used a 2×2 contingency table. The entries in the four cells were as follows: a (HAM/TSP, positive for protective genotype) = 183, b (HAM/TSP, negative for protective genotype) = 47, c (AC, positive for protective genotype) = 181, d (AC, negative for protective genotype) = 21. The fraction (F_p_) of potential cases of HAM/TSP in the population that is prevented by the protective genotype is given by F_p_ = (1−R)×[1−(d×r_1_/b×r_2_)], where R = prevalence rate of HAM/TSP in the population (estimated as 1% of the HTLV-1-infected population), r_1_ = a+b and r_2_ = c+d. F_p_ is approximately normally distributed: the standard deviation is given by SD (F_p_) = (1−R−F_p_)×√[(c/d×r_2_)+(a/b×r_1_)].

### Detection of HTLV-1-specific CD8^+^ T cells

Peripheral blood mononuclear cells (PBMC) were isolated from whole blood from HTLV-1 infected individuals by density gradient centrifugation.

#### IFNγ ELISpot

PBMC were depleted of CD4^+^ T cells using MACS beads (Miltenyi Biotec). The resulting cells were cultured in duplicate at a density of 100,000 cells per well in the presence of a range of concentrations of pooled overlapping 20mer peptides (offset by 6 amino acids) spanning HBZ, Tax, or with medium alone. After 6 hours, IFN-γ producing cells were detected by ELISpot (Mabtech). The threshold for a positive response to peptide was defined as greater than the mean plus two standard deviations of the number of spots in the medium only control.

#### CD107 mobilisation assay

1×10^6^ PBMC were cultured in a 400 µl volume in the presence of 5 µl anti-CD107a-PE (eBioscience, CA) 1.4 µg/ml monensin (eBioscience), 20 µg/ml DNase (Sigma Aldrich, UK) with 2 µM HBZ peptide pool (Mimotopes, Australia), or the equivalent volume of peptide solvent, acetonitrile. After 5h, the cells were harvested and stained for 30 min with Live/Dead red (Invitrogen,CA), a fixable viability stain. Cells were fixed and permeablised using ebioscience FoxP3 staining buffer set according to the manufacturer's instructions, then stained with anti-CD3-APC-eFluor780, anti-CD4-eFluor450, anti-CD8-PECy5 (all eBioscience) anti-IFN-γ-FITC, anti-CD14-ECD, anti-CD19-ECD (all Beckman Coulter, France). Samples analysed by flow cytometry using a Cyan ADP (Beckman Coulter), and summit software (DAKO). Doublets, dead cells, monocytes and B cells were excluded from the analysis on the basis of forward and side scatter, pulse width, viability staining, and CD14 or CD19 expression. Antigen-specific CD8^+^ T cells were identified as CD3^+^CD8^+^ cells capable of producing IFN-γ and/or mobilising CD107 to the cell surface.

### CD8^+^ lysis assay: chromium release

PBMC from HLA A*0201^+^ and HLA A*0201^−^ HTLV-1 infected individuals were depleted of CD8^+^ cells, then enriched for CD25^+^ cells using MACS beads (Miltenyi Biotech, Germany), according to manufacturer's instructions. CD25^+^ cells were cultured for 16h to allow for viral antigen expression and presentation, then labelled with ^51^Cr by incubating for 1h in the presence of 50–100 µCi Na_2_CrO_4_ (MP Biomedicals, USA). Labelled cells were washed extensively and placed in culture in triplicate (40,000 cells/well) in the presence of defined ratios of HBZ-1, a CTL clone which recognises HBZ 26–34 in the context of HLA A*0201 [37], alone or in the presence of 5% Triton x-100 (Sigma Aldrich). As a control, ^51^Cr labelled B-LCL (autologous to the CTL clone) were cultured at the same ratios, with and without 1 µM HBZ 26–34 peptide. After 4h, culture supernatants were harvested, placed on a scintillation plate, and ^51^Cr release was assayed using a beta counter. Total specific lysis was calculated using the following formula: [chromium release (test well)−chromium release (no CTL control)]/ [chromium release (Triton−100% lysis)−chromium release (no CTL control)]*100, expressed as a percentage specific lysis of total cells. As not all CD4^+^CD25^+^T cells are infected, and thus do not represent targets for the CTL line, an estimate of specific lysis of infected cells was also calculated, making the conservative assumption that all the viral load is present in CD25^+^ cells, [57], and that the CTL line only kills infected cells. Percentage infected cells lysed was calculated using the following formula: [Percentage total cells lysed]/[fraction of CD4^+^CD25^+^ cells that are infected i.e. provirus positive].

### HTLV-1 proteome

The reference strain is from [58], with the exception of HBZ, which was identified more recently and described in [35] (Supporting Information S1: HTLV-1 reference strain).

## Supporting Information

Supporting Information S1 Supporting information
(0.78 MB DOC)

## References

[1] Vine AM, Heaps AG, Kaftantzi L, Mosley A, Asquith B (2004). The role of CTLs in persistent viral infection: cytolytic gene expression in CD8+ lymphocytes distinguishes between individuals with a high or low proviral load of human T cell lymphotropic virus type 1.. J Immunol.

[2] Asquith B, Mosley AJ, Barfield A, Marshall SEF, Heaps A (2005). A functional CD8+ cell assay reveals individual variation in CD8+ cell antiviral efficacy and explains differences in human T-lymphotropic virus type 1 proviral load.. J Gen Virol.

[3] Jeffery KJ, Usuku K, Hall SE, Matsumoto W, Taylor GP (1999). HLA alleles determine human T-lymphotropic virus-I (HTLV-I) proviral load and the risk of HTLV-I-associated myelopathy.. Proc Natl Acad Sci U S A.

[4] Jacobson S (2002). Immunopathogenesis of human T cell lymphotropic virus type I-associated neurologic disease.. J Infect Dis.

[5] Kubota R, Kawanishi T, Matsubara H, Manns A, Jacobson S (2000). HTLV-I specific IFN-gamma+ CD8+ lymphocytes correlate with the proviral load in peripheral blood of infected individuals.. J Neuroimmunol.

[6] Jacobson S, Shida H, McFarlin DE, Fauci AS, Koenig S (1990). Circulating CD8+ cytotoxic T lymphocytes specific for HTLV-I pX in patients with HTLV-I associated neurological disease.. Nature.

[7] Greten TF, Slansky JE, Kubota R, Soldan SS, Jaffee EM (1998). Direct visualization of antigen-specific T cells: HTLV-1 Tax11-19- specific CD8(+) T cells are activated in peripheral blood and accumulate in cerebrospinal fluid from HAM/TSP patients.. Proc Natl Acad Sci U S A.

[8] Sundaram R, Sun Y, Walker CM, Lemonnier FA, Jacobson S (2003). A novel multivalent human CTL peptide construct elicits robust cellular immune responses in HLA-A*0201 transgenic mice: implications for HTLV-1 vaccine design.. Vaccine.

[9] Kannagi M, Harada S, Maruyama I, Inoko H, Igarashi H (1991). Predominant recognition of human T cell leukemia virus type I (HTLV-I) pX gene products by human CD8+ cytotoxic T cells directed against HTLV-I-infected cells.. Int Immunol.

[10] Goon PK, Biancardi A, Fast N, Igakura T, Hanon E (2004). Human T cell lymphotropic virus (HTLV) type-1-specific CD8+ T cells: frequency and immunodominance hierarchy.. J Infect Dis.

[11] Jeffery KJ, Usuku K, Hall SE, Matsumoto W, Taylor GP (1999). HLA alleles determine human T-lymphotropic virus-I (HTLV-I) proviral load and the risk of HTLV-I-associated myelopathy.. Proc Natl Acad Sci U S A.

[12] Parker CE, Nightingale S, Taylor GP, Weber J, Bangham CR (1994). Circulating anti-Tax cytotoxic T lymphocytes from human T-cell leukemia virus type I-infected people, with and without tropical spastic paraparesis, recognize multiple epitopes simultaneously.. J Virol.

[13] Hausmann S, Biddison WE, Smith KJ, Ding YH, Garboczi DN (1999). Peptide recognition by two HLA-A2/Tax11-19-specific T cell clones in relationship to their MHC/peptide/TCR crystal structures.. J Immunol.

[14] Baalen CA, Guillon C, Baalen Mv M, Verschuren EJ, Boers PH (2002). Impact of antigen expression kinetics on the effectiveness of HIV-specific cytotoxic T lymphocytes.. Eur J Immunol.

[15] Niewiesk S, Daenke S, Parker CE, Taylor G, Weber J (1994). The transactivator gene of human T-cell leukemia virus type I is more variable within and between healthy carriers than patients with tropical spastic paraparesis.. J Virol.

[16] Barber DL, Wherry EJ, Masopust D, Zhu B, Allison JP (2006). Restoring function in exhausted CD8 T cells during chronic viral infection.. Nature.

[17] Lichterfeld M, Yu XG, Mui SK, Williams KL, Trocha A (2007). Selective depletion of high-avidity human immunodeficiency virus type 1 (HIV-1)-specific CD8+ T cells after early HIV-1 infection.. J Virol.

[18] Streeck H, Brumme ZL, Anastario M, Cohen KW, Jolin JS (2008). Antigen load and viral sequence diversification determine the functional profile of HIV-1-specific CD8+ T cells.. PLoS Med.

[19] Bangham CR (2009). CTL quality and the control of human retroviral infections.. Eur J Immunol.

[20] Gaudray G, Gachon F, Basbous J, Biard-Piechaczyk M, Devaux C (2002). The complementary strand of the human T-cell leukemia virus type 1 RNA genome encodes a bZIP transcription factor that down-regulates viral transcription.. J Virol.

[21] Kubota R, Hanada K, Furukawa Y, Arimura K, Osame M (2007). Genetic stability of human T lymphotropic virus type I despite antiviral pressures by CTLs.. J Immunol.

[22] Pique C, Connan F, Levilain JP, Choppin J, Dokhélar MC (1996). Among all human T-cell leukemia virus type 1 proteins, tax, polymerase, and envelope proteins are predicted as preferential targets for the HLA-A2-restricted cytotoxic T-cell response.. J Virol.

[23] Sundaram R, Sun Y, Walker CM, Lemonnier FA, Jacobson S (2003). A novel multivalent human CTL peptide construct elicits robust cellular immune responses in HLA-A*0201 transgenic mice: implications for HTLV-1 vaccine design.. Vaccine.

[24] Goon PKC, Biancardi A, Fast N, Igakura T, Hanon E (2004). Human T cell lymphotropic virus (HTLV) type-1-specific CD8+ T cells: frequency and immunodominance hierarchy.. J Infect Dis.

[25] Borghans JAM, Mølgaard A, de Boer RJ, Kesmir C (2007). HLA Alleles Associated with Slow Progression to AIDS Truly Prefer to Present HIV-1 p24.. PLoS ONE.

[26] Vider-Shalit T, Almani M, Sarid R, Louzoun Y (2009). The HIV hide and seek game: an immunogenomic analysis of the HIV epitope repertoire.. Aids.

[27] Vider-Shalit T, Sarid R, Maman K, Tsaban L, Levi R (2009). Viruses selectively mutate their CD8+ T-cell epitopes–a large-scale immunomic analysis.. Bioinformatics.

[28] Hoof I, Kesmir C, Lund O, Nielsen M (2008). Humans with chimpanzee-like major histocompatibility complex-specificities control HIV-1 infection.. Aids.

[29] Schellens IM, Kesmir C, Miedema F, van Baarle D, Borghans JA (2008). An unanticipated lack of consensus cytotoxic T lymphocyte epitopes in HIV-1 databases: the contribution of prediction programs.. Aids.

[30] Schmid BV, Kesmir C, de Boer RJ (2009). The distribution of CTL epitopes in HIV-1 appears to be random, and similar to that of other proteomes.. BMC Evol Biol.

[31] Kosmrlj A, Read EL, Qi Y, Allen TM, Altfeld M Effects of thymic selection of the T-cell repertoire on HLA class I-associated control of HIV infection.. Nature.

[32] Jeffery KJ, Siddiqui AA, Bunce M, Lloyd AL, Vine AM (2000). The influence of HLA class I alleles and heterozygosity on the outcome of human T cell lymphotropic virus type I infection.. J Immunol.

[33] Nagai M, Usuku K, Matsumoto W, Kodama D, Takenouchi N (1998). Analysis of HTLV-I proviral load in 202 HAM/TSP patients and 243 asymptomatic HTLV-I carriers: high proviral load strongly predisposes to HAM/TSP.. J Neurovirol.

[34] Vine AM, Witkover AD, Lloyd AL, Jeffery KJM, Siddiqui A (2002). Polygenic control of human T lymphotropic virus type I (HTLV-I) provirus load and the risk of HTLV-I-associated myelopathy/tropical spastic paraparesis.. J Infect Dis.

[35] Satou Y, Yasunaga J-i, Yoshida M, Matsuoka M (2006). HTLV-I basic leucine zipper factor gene mRNA supports proliferation of adult T cell leukemia cells.. Proc Natl Acad Sci U S A.

[36] Matsuoka M, Green P (2009). The HBZ gene, a key player in HTLV-1 pathogenesis.. Retrovirology.

[37] Suemori K, Fujiwara H, Ochi T, Ogawa T, Matsuoka M (2009). HBZ is an immunogenic protein, but not a target antigen for human T-cell leukemia virus type 1-specific cytotoxic T lymphocytes.. J Gen Virol.

[38] Usui T, Yanagihara K, Tsukasaki K, Murata K, Hasegawa H (2008). Characteristic expression of HTLV-1 basic zipper factor (HBZ) transcripts in HTLV-1 provirus-positive cells.. Retrovirology.

[39] Saito M, Matsuzaki T, Satou Y, Yasunaga J-I, Saito K (2009). In vivo expression of the HBZ gene of HTLV-1 correlates with proviral load, inflammatory markers and disease severity in HTLV-1 associated myelopathy/tropical spastic paraparesis (HAM/TSP).. Retrovirology.

[40] Basbous J, Arpin C, Gaudray G, Piechaczyk M, Devaux C (2003). The HBZ factor of human T-cell leukemia virus type I dimerizes with transcription factors JunB and c-Jun and modulates their transcriptional activity.. J Biol Chem.

[41] Li M, Kesic M, Yin H, Yu L, Green PL (2009). Kinetic Analysis of Human T-cell Leukemia Virus Type 1 Gene Expression in Cell Culture and Infected Animals.. J Virol.

[42] Azuma T, Otsuki T, Kuzushima K, Froelich CJ, Fujita S (2004). Myeloma cells are highly sensitive to the granule exocytosis pathway mediated by WT1-specific cytotoxic T lymphocytes.. Clin Cancer Res.

[43] Jiang SB, Ojcius DM, Persechini PM, Young JD (1990). Resistance of cytolytic lymphocytes to perforin-mediated killing. Inhibition of perforin binding activity by surface membrane proteins.. J Immunol.

[44] Muller C, Tschopp J (1994). Resistance of CTL to perforin-mediated lysis. Evidence for a lymphocyte membrane protein interacting with perforin.. J Immunol.

[45] Larsen MV, Lundegaard C, Lamberth K, Buus S, Brunak S (2005). An integrative approach to CTL epitope prediction: a combined algorithm integrating MHC class I binding, TAP transport efficiency, and proteasomal cleavage predictions.. Eur J Immunol.

[46] Buus S, Lauemøller SL, Worning P, Kesmir C, Frimurer T (2003). Sensitive quantitative predictions of peptide-MHC binding by a ‘Query by Committee’ artificial neural network approach.. Tissue Antigens.

[47] Nielsen M, Lundegaard C, Worning P, Lauemøller SL, Lamberth K (2003). Reliable prediction of T-cell epitopes using neural networks with novel sequence representations.. Protein Sci.

[48] Macnamara A, Kadolsky U, Bangham CRM, Asquith B (2009). T-Cell Epitope Prediction: Rescaling Can Mask Biological Variation between MHC Molecules.. PLoS Comput Biol.

[49] Heckerman D, Kadie C, Listgarten J (2007). Leveraging information across HLA alleles/supertypes improves epitope prediction.. J Comput Biol.

[50] Peters B, Bui H-H, Frankild S, Nielson M, Lundegaard C (2006). A community resource benchmarking predictions of peptide binding to MHC-I molecules.. PLoS Comput Biol.

[51] Schmid B, Kesmir C, de Boer RJ (2008). The specificity and polymorphism of the MHC class I prevents the global adaptation of HIV-1 to the monomorphic proteasome and TAP.. PLoS ONE.

[52] Fortier M-H, Caron E, Hardy M-P, Voisin G, Lemieux S (2008). The MHC class I peptide repertoire is molded by the transcriptome.. J Exp Med.

[53] Borghans JAM, Mølgaard A, de Boer RJ, Kesmir C (2007). HLA Alleles Associated with Slow Progression to AIDS Truly Prefer to Present HIV-1 p24.. PLoS ONE.

[54] R Development Core Team (2008).

[55] Crawley MJ (2007).

[56] Kaslow RA, McNicholl J, Hill AVS (2008). Genetic susceptibility to infectious diseases.

[57] Yamano Y, Cohen CJ, Takenouchi N, Yao K, Tomaru U (2004). Increased expression of human T lymphocyte virus type I (HTLV-I) Tax11-19 peptide-human histocompatibility leukocyte antigen A*201 complexes on CD4+ CD25+ T Cells detected by peptide-specific, major histocompatibility complex-restricted antibodies in patients with HTLV-I-associated neurologic disease.. J Exp Med.

[58] Seiki M, Hattori S, Hirayama Y, Yoshida M (1983). Human adult T-cell leukemia virus: complete nucleotide sequence of the provirus genome integrated in leukemia cell DNA.. Proc Natl Acad Sci U S A.

