# Supplementary material for: HLA Class I Binding of HBZ Determines Outcome in HTLV-1 Infection
Source: PLoS Pathog. 2010 Sep 23;6(9):e1001117. doi: 10.1371/journal.ppat.1001117 (PMC2944806; doi:10.1371/journal.ppat.1001117)
Supplement: Supporting Information S1 — Supporting information (0.78 MB DOC) [file ppat.1001117.s001.doc]

Supporting Information S1

Table S1: The experimentally measured binding affinity of 200 HTLV-I peptides. Note the binding score is with respect to a reference good binding peptide for each allele and therefore scores can be compared within an allele but not between alleles.

| A*0201 | | B*0702 | | A*2402 | | B*3501 | |
| --- | --- | --- | --- | --- | --- | --- | --- |
| Peptide | Score | Peptide | Score | Peptide | Score | Peptide | Score |
| ASGLFRCLP | 2.99 | ASGLFRCLP | 2 | ASGLFRCLP | 4.14 | ASGLFRCLP | 0 |
| AVLDGLLSL | 92.9 | AVLDGLLSL | 18.76 | AVLDGLLSL | 1.41 | AVLDGLLSL | 0 |
| GFGQSLLFG | 0.29 | GFGQSLLFG | 0 | GFGQSLLFG | 1.06 | GFGQSLLFG | 0 |
| IQYSSFHSL | 93.93 | IQYSSFHSL | 2.94 | IQYSSFHSL | 87.52 | KALMPVFTL | 11.5 |
| KALMPVFTL | 79.88 | KALMPVFTL | 40.22 | KALMPVFTL | 8.05 | SAQWIPWRL | 14.94 |
| RICPINYSL | 71.46 | RICPINYSL | 110.59 | RICPINYSL | 77.87 | AAHHWLNFL | 0 |
| SAQWIPWRL | 24.03 | SAQWIPWRL | 0 | SAQWIPWRL | 27.07 | FVERLNIAL | 13.69 |
| AAHHWLNFL | 5.86 | AAHHWLNFL | 27.59 | HQLKKFLKI | 46.49 | RVNEILHIL | 8.02 |
| FVERLNIAL | 6.6 | FVERLNIAL | 104.14 | IALETPARI | 9.68 | SLVQLRQAL | 0 |
| KISLTTGAL | 3.54 | KISLTTGAL | 71.26 | LSPPITWPL | 4.52 | LSPPITWPL | 41.83 |
| LVLQSSSFI | 41.33 | LVLQSSSFI | 1.65 | STLTTPGLI | 0 | ELVDGLLSL | 0 |
| RVNEILHIL | 49.35 | RVNEILHIL | 89.83 | WQMKDLQAI | 0 | LLQEKEDLM | 0 |
| SLVQLRQAL | 6.84 | SLVQLRQAL | 119.22 | LAAHHWLNF | 35.91 | LTPPITHTT | 0 |
| HQLKKFLKI | 0 | LAAHHWLNF | 4.59 | QAMRKYSPF | 56.78 | LVEELVDGL | 0 |
| IALETPARI | 17.01 | QAMRKYSPF | 140.22 | RVIGSALQF | 65.17 | YILWDKQIL | 0 |
| LSPPITWPL | 34.53 | RVIGSALQF | 87.63 | TFLKTAAPL | 86.12 | YISQDFLNM | 0 |
| STLTTPGLI | 0.84 | TFLKTAAPL | 28.15 | AWQNGLLPF | 92.6 | LAAHHWLNF | 75.12 |
| WQMKDLQAI | 44.68 | AASGLFRCL | 107.28 | DLMGEVNYW | 62.28 | QAMRKYSPF | 69.18 |
| AAGAALIPV | 34.35 | APLPHTSQC | 135.12 | EKAVLDGLL | 0 | RVIGSALQF | 42.94 |
| ALLGEIQWV | 84.64 | DPISRLNAL | 115.84 | EYLKRKEEE | 0 | AASGLFRCL | 7.33 |
| ELVDGLLSL | 25.11 | FPGFGQSLL | 102.99 | EYQQLWLAA | 2.7 | DPISRLNAL | 67.38 |
| FMQTIRLAV | 95.61 | HPGQLGAFL | 60.82 | GYPGRVNEI | 82.68 | FPGFGQSLL | 55.31 |
| GIDGYTRQL | 28.99 | KARRRRRAE | 0 | GYTRQLEGE | 35.37 | HPGQLGAFL | 34.24 |
| GLLSLEEEL | 63.68 | LEAERRKLL | 1.08 | ICPINYSLL | 69.19 | LPFHSTLTT | 102.24 |
| ILIQTQAQI | 79.21 | LPFHSTLTT | 97.77 | IFSRSASPI | 62.41 | LPVSCPEDL | 74.86 |
| ILPEDCLPT | 15.96 | LPGLNSRQW | 6.78 | IWQGDITHF | 64.83 | MPVFTLSPV | 114.77 |
| KLLQEKEDL | 0 | LPVMHPHGA | 9.38 | KQIAEYLKR | 3.56 | QPARAPVTL | 55.56 |
| LLFGYPVYV | 24.95 | LPVSCPEDL | 6.44 | KYKNTLYRL | 124.9 | RPPRGLAAH | 34.86 |
| LLITPVLQL | 114.87 | MPVFTLSPV | 142.96 | KYLYHYLRT | 2.63 | YPGRVNEIL | 109.69 |
| LLLDLPADI | 77.78 | PPNHRPWQM | 103.11 | KYTLQSYGL | 104.78 | NFLQAAYRL | 3.16 |
| LLQEKEDLM | 46.36 | QPARAPVTL | 135.43 | LWLAAFAAL | 30.63 | YWQGRLEAM | 7.1 |
| LLQYLCSSL | 45.68 | QPRPPPGPC | 138.82 | NFLQAAYRL | 113.8 | CPINYSLLA | 102.68 |
| LLYKISLTT | 78.05 | RAEKKAADV | 2.78 | NYSLLASLL | 105.08 | CPLCQDPTH | 36.7 |
| LMGEVNYWQ | 12.98 | RDRQRRAEE | 1.54 | PYHAFVERL | 113.04 | DPILRSLAY | 72.18 |
| LQYLCSSLV | 47.17 | RGRLRRGPP | 1.21 | PYKRIEELL | 99.06 | EEEKQIAEY | 8.99 |
| LTPPITHTT | 31.05 | RPAPPPPSS | 93.08 | PYNPTSSGL | 104.86 | EPEEDALLL | 11.92 |
| LVEELVDGL | 2.64 | RPPRGLAAH | 143.59 | QYLCSSLVA | 4.85 | EPEPEEDAL | 28.71 |
| QLDSLISEA | 52.07 | RRRAEKKAA | 0 | SFHSLHLLF | 107.68 | EPGPSSYDF | 102.27 |
| QLEGEVESL | 18.58 | SARLHRHAL | 236.66 | SFLLSHGLI | 79.27 | FPQCTILQY | 103.38 |
| QLGAFLTNV | 80.99 | TPKDKTKVL | 113.7 | SWASILQGL | 123.67 | FPTQRTSKT | 81.35 |
| QLLASAVLL | 100.23 | TPNIPPSFL | 138.52 | SYGLLCQTI | 114.37 | LASLLPKGY | 98.24 |
| QLWLAAFAA | 64.37 | VPIRSRWAL | 125.64 | TTPGLIWTF | 215.15 | LPEDCLPTT | 52.2 |
| TLGQHLPTL | 54.56 | VPYKRIEEL | 133.31 | TTPNIPPSF | 0.43 | LPTTLFQPA | 119.5 |
| TLSFPDPGL | 0 | YPGRVNEIL | 158.86 | TWPLLPHVI | 118.19 | MQELGIDGY | 48.1 |
| TLTAWQNGL | 54.64 | Intermediate | 43.52 | VFTLSPVII | 97.21 | QAAPGSPQF | 86.5 |
| TLYRLHVWV | 73.87 | Positive | 100 | VLQSSSFIF | 82.42 | WALPELQAL | 73.45 |
| YILWDKQIL | 39 |  |  | VSCPEDLLV | 9.6 | WPLLPHVIF | 49.66 |
| YISQDFLNM | 10.48 |  |  | WQGRLEAMW | 2.54 | WTFTDGTPM | 137.29 |
| YLCSSLVAS | 42.17 |  |  | YWQGRLEAM | 40.32 | WTINHLNVL | 1.04 |
| YLYQLSPPI | 132.52 |  |  | Intermediate | 30.79 | Intermediate | 96.47 |
| Intermediate | 29.56 |  |  | Positive | 100 | Positive | 100 |
| Positive | 100 |  |  |  |  |  |  |

Table S2: Comparison of the strength of binding of the alleles from AC and HAM/TSP patients to each of the 12 HTLV-I proteins. Significance after correction classifies the *P* values after Bonferroni’s correction for multiple comparisons was applied: < 0.05 *, < 0.01 **, < 0.001 ***.

| **Protein** | **Metaserver** | | | **Epipred** | | |
| --- | --- | --- | --- | --- | --- | --- |
|  | **P value**  **(2 tailed)** | **Group with strongest binding** | **Significance after correction** | **P value**  **(2 tailed)** | **Group with strongest binding** | **Significance after correction** |
| pol | 0.0005 | AC | ** | 0.0715 | AC | - |
| env | 0.0019 | HAM | * | 0.8308 | HAM | - |
| rof | 0.0023 | HAM | * | 0.1441 | HAM | - |
| tax | 0.3320 | AC | - | 0.0505 | HAM | - |
| p12 | 0.0168 | HAM | - | 0.2232 | HAM | - |
| rex | 0.4706 | AC | - | 0.7410 | HAM | - |
| **HBZ** | **0.0002** | **AC** | ****** | **0.000002** | **AC** | ******* |
| gag | 0.0011 | AC | * | 0.0366 | HAM | - |
| pro | 0.0970 | HAM | - | 0.7077 | HAM | - |
| tof | 0.4111 | HAM | - | 0.0005 | HAM | - |
| p13 | 0.8524 | AC | - | 0.5065 | HAM | - |
| p21 | 0.0341 | AC | - | 0.0004 | HAM | * |

Table S3: The significant predictors and their associated P values for each of the multiple regression models of proviral load

|  | Protein Binding (A and B only) | | | Genotype (A and B only) | | |
| --- | --- | --- | --- | --- | --- | --- |
| AC Proviral Load | HBZ | 0.001 | *** | A*02 | 0.01 | ** |
|  | Pro | 0.013 | * |  |  |  |
|  | R^2^ = 0.054 | | | R^2^ = 0.034 | | |
| HAM/TSP Proviral Load | HBZ | 0.017 | * | B*54 | 0.019 | * |
|  | R^2^ = 0.026 | | | R^2^ = 0.025 | | |

Table S4: An example of the rank method used to measure the targeting of specific HTLV-I proteins by HLA class-I alleles. The predicted binding affinities for every overlapping nonamer peptide in the HTLV-I proteome (N =3389) was derived for each allele of interest. These were then ordered from strongest to weakest binding for each allele. Then, for each protein, the associated rank values were taken as a measure of the strength of binding of that protein by that allele. In the table below, the alleles previously associated with disease outcome and proviral load are shown along with the ordered HTLV-I peptides that bind to that allele (1 being the strongest, 3389 being the weakest). For example, the strongest binders from Tax to A*0201 would be ranked {1, 2, …} , the strongest binders from Pol to A*0201 would be {3, 4, 5, 7, …}.

|  | Cw*0801 | | B*5401 | | A*0201 | |
| --- | --- | --- | --- | --- | --- | --- |
| 1 | Gag | TPKDKTKVL | Tax | LPTTLFQPA | Tax | YLYQLSPPI |
| 2 | Pol | PADPKEKDL | Pro | LPVIPLDPA | Tax | LLFGYPVYV |
| 3 | Rof | RPPPAPCLL | Env | FPFSLLVDA | Pol | ALLGEIQWV |
| 4 | P12 | RPPPAPCLL | Pol | MPVFTLSPV | Pol | SLISHGLPV |
| 5 | Gag | NANKECQKL | Rof | LPITMRFPA | Pol | FQPYFAFTV |
| 6 | Gag | ANNPQQQGL | P12 | LPITMRFPA | Gag | FMQTIRLAV |
| 7 | Gag | GAPPNHRPW | Pro | LPFRTTPIV | Pol | LTYDAVPTV |
| … | … | … | … | … | … | … |
| 3389 | P12 | LLLFLLPPS | Tax | DNDHEPQIS | Tax | DNDHEPQIS |

Each test then uses these rank values as follows:

1. *Protective class I alleles bind HBZ strongly:* For each protein (in this example – Tax), the ranks of the top 8 binding peptides *from the protein to the allele* were compared between detrimental (B*5401) and protective (A*0201 and Cw*0801) alleles

| Count |  | Cw*0801 Ranks |  | A*0201 Ranks |  | B*5401 Ranks |
| --- | --- | --- | --- | --- | --- | --- |
| 1 |  | 17 |  | 1 |  | 1 |
| 2 |  | 18 |  | 2 |  | 17 |
| 3 |  | 26 |  | 14 |  | 28 |
| 4 |  | 55 | **AND** | 23 | **VERSUS** | 31 |
| 5 |  | 90 |  | 33 |  | 32 |
| 6 |  | 92 |  | 35 |  | 33 |
| 7 |  | 95 |  | 46 |  | 39 |
| 8 |  | 104 |  | 67 |  | 40 |

1. *Asymptomatic carriers bind HBZ more strongly than HAM/TSP patients:* For each protein (in this example – HBZ), the rank of the strongest binding peptide to each allele of the A and B loci was found for each individual. These were then compared between HAM/TSP and AC. Key = {allele, rank of strongest binding peptide from the protein of interest to that allele}

HAM/TSP

| Individual | Rank of strongest binding HBZ peptide to locus A1 | Rank of strongest binding HBZ peptide to locus A2 | Rank of strongest binding HBZ peptide to locus B1 | Rank of strongest binding HBZ peptide to locus B2 |
| --- | --- | --- | --- | --- |
| HAM/TSP 1 | {A2402, **208**} | {A2402, **208**} | {B4002, **3**} | {B4002, **3**} |
| HAM/TSP 2 | {A2402, **208**} | {A3101, **42**} | {B5101, **42**} | {B0702, **84**} |
| HAM/TSP 3 | {A2402, **208**} | {A2601, **2**} | {B5401, **125**} | {B3501, **93**} |
| … | … | … | … | … |
| HAM/TSP 230 | {A2601, **2**} | {A3101, **42**} | {B3501, **93**} | {B3501, **93**} |

AC

| Individual | Rank of strongest binding HBZ peptide to HLA-A1 | Rank of strongest binding HBZ peptide to locus A2 | Rank of strongest binding HBZ peptide to locus B1 | Rank of strongest binding HBZ peptide to locus B2 |
| --- | --- | --- | --- | --- |
| AC 1 | {A2402, **208**} | {A2601, **2**} | {B5401, **125**} | {B5601, **NA**} |
| AC 2 | {A2402, **208**} | {A3301, **9**} | {B3501, **93**} | {B4402, **2**} |
| AC 3 | {A2402, **208**} | {A2402, **208**} | {B3501, **93**} | {B4402, **2**} |
| … | … | … | … | … |
| AC 202 | {A0201, **22**} | A3101, **42**} | {B3501, **93**} | {B4001, **7**} |

All rank values (in bold) for the HAM/TSP group were compared against all rank values (in bold) for the AC group using a Wilcoxon-Mann-Whitney test

1. *HBZ peptide binding is a consistent predictor of proviral load:* Again, for each protein (in this example – HBZ), the relationship between the number of strong binding alleles to peptides from that protein and proviral load was examined. For each protein, the definition of a strong binding allele to *that* protein was as follows: For HBZ, the {HLA, rank} data from *both* HAM/TSP and AC groups was ordered from strongest to weakest binding…

| 1 | 2 | 3 | 4 | 5 | 6 |
| --- | --- | --- | --- | --- | --- |
| {A2601, 2} | {A2601, 2} | {A2601, 2} | {B4402, 2} | {B4402, 2} | {B4002, 3} |
|  |  |  |  |  |  |
| 7 | 8 | 9 | 10 | … | *n* = number of alleles in the cohort (1728) |
| {B4002, 3} | {B4001, 7} | {A3301, 9} | {A0201, 22} | … | {A2402, 208} |

…any allele contained within the top 40% of this list was defined as a strong binding allele to peptides from that protein.

Table S5: Results of hypothesis testing repeated using different epitope prediction methods (Metaserver and Epipred) and different metrics (a rank measure which only compares within alleles (i.e. not between alleles) and a raw binding score measure which compares between as well as within alleles).

|  | Null hypothesis | Rank measure | | Raw score | | Conclusion |
| --- | --- | --- | --- | --- | --- | --- |
|  |  | Metaserver | Epipred | Metaserver | Epipred |  |
| 1 | Protective and detrimental alleles target HBZ equally | 0.0002 | | _[[1]](#footnote-2) | _^1^ | Protective alleles bind HBZ significantly more strongly than detrimental alleles |
| 2 | AC and HAM/TSP patients target HBZ equally | 0.0002 | 0.000002 | 0.002 | 0.001 | ACs have HLA alleles that bind HBZ significantly more strongly compared to HAM/TSP patients |
| 3 | AC and HAM/TSP patients target HBZ equally [excluding A02, B54 and Cw08] | 0.04 | 0.006 | 0.14 | 0.03 | ACs bind HBZ significantly more strongly compared to HAM/TSP patients even when known protective and detrimental alleles are excluded |
| 4 | There is no correlation between proviral load and the number of alleles that bind HBZ strongly | 0.016 | 0.1 | 0.01 | 0.032 | The higher the number of strong binding alleles to HBZ per individual, the lower their proviral load |
| 5 | There is no correlation between proviral load and the strength of HBZ binding | 0.008 | 0.04 | 0.003 | 0.085 | The greater the strength of HBZ binding (rank method), the lower the proviral load |
| 6 | There is no correlation between load reduction (count) and disease prevalence reduction | 0.0005 | 0.02 | 0.004 | 0.03 | Proteins that are strongly bound by asymptomatic carriers are, independently, those associated with a greater reduction in load when bound |
| 7 | There is no correlation between load reduction (rank) and disease prevalence reduction | < 2.2*10^-16^ | 0.003 | 0.002 | 0.2 | As above, using the rank measure to quantify the effect of binding strength on proviral load |


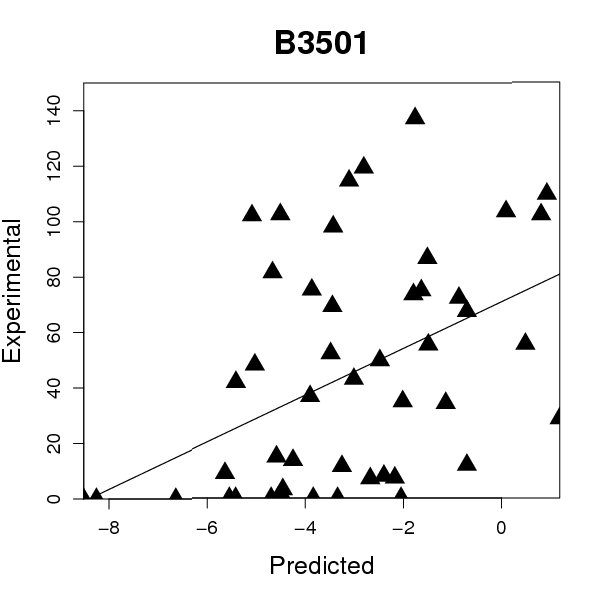


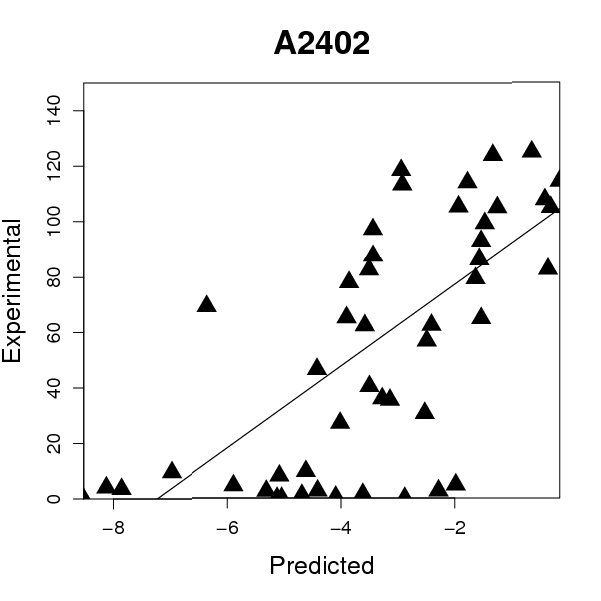

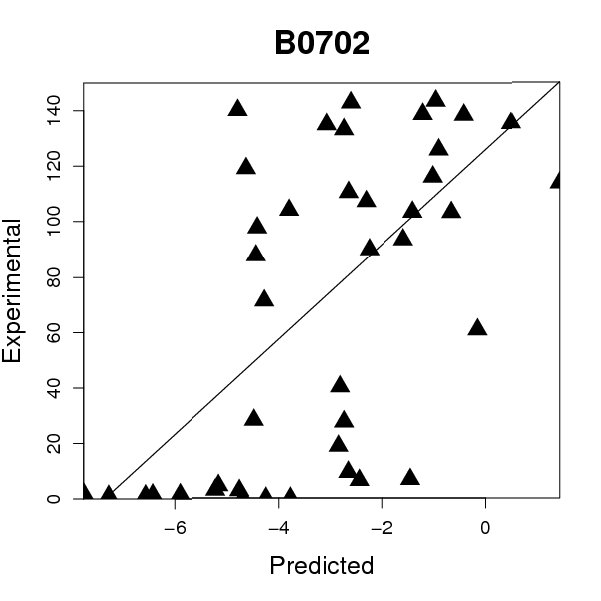


**Figure S1: The correlation between the experimentally measured binding affinities (% of control peptide) and the predicted binding affinities from Epipred for each of the 4 alleles analysed.** The measured affinities are given in Table S1 in Supporting Information S1. A*0201: R_S_=0.48, p=4x10^-4^; B*0702: R_S_=0.65, p=2x10^-6^; A*2402: R_S_=0.68, p=8x10^-8^; B*3501: R_S_=0.47, p=6x10^-4^.


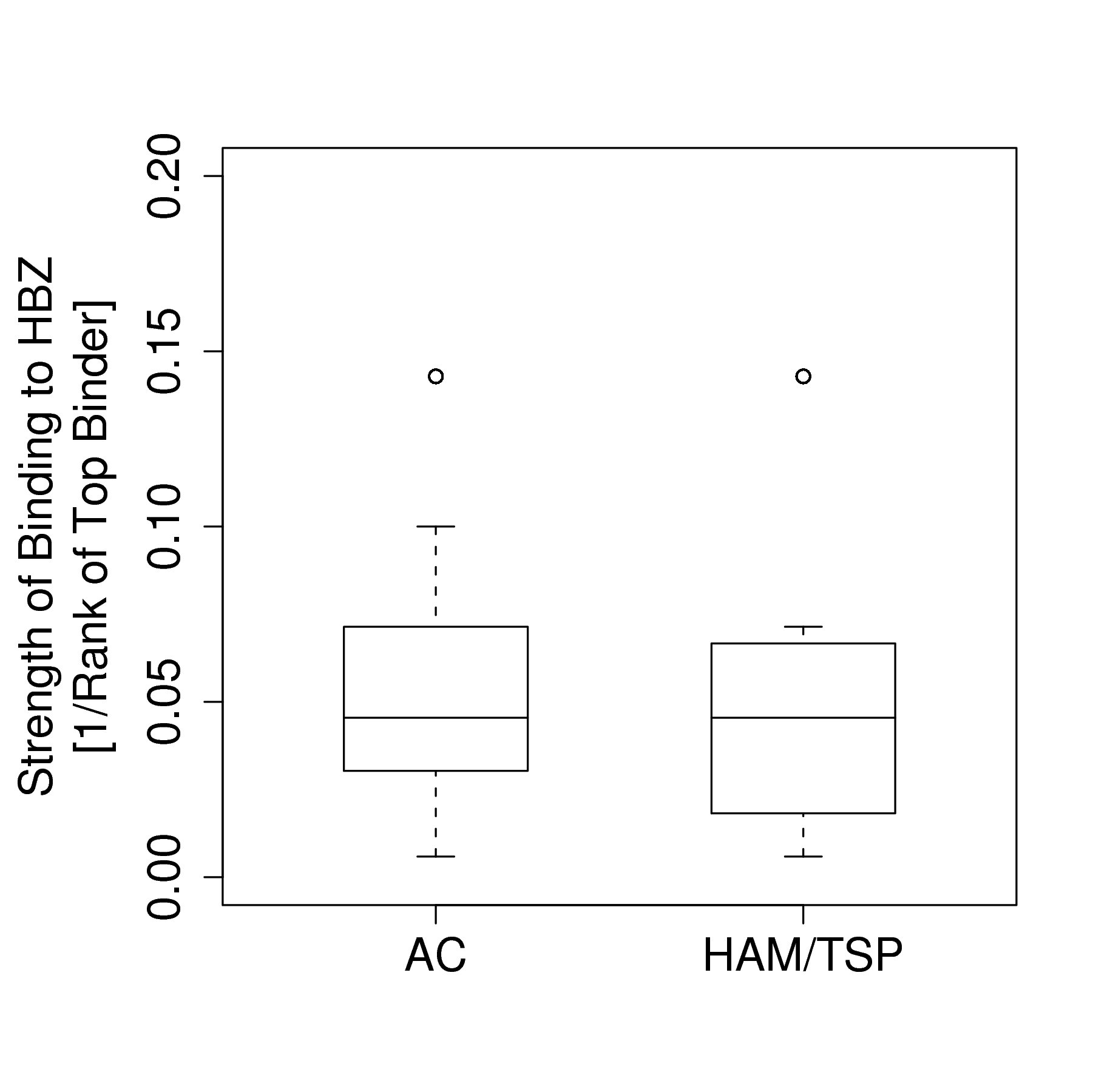


**Figure S2:** A comparison of the strength of binding to HBZ (the reciprocal of the ranks of the top-binding peptides) of the alleles of the AC group and the HAM/TSP group of the Kagoshima cohort (p=0.000002). Epitope binding predictions made using Epipred.


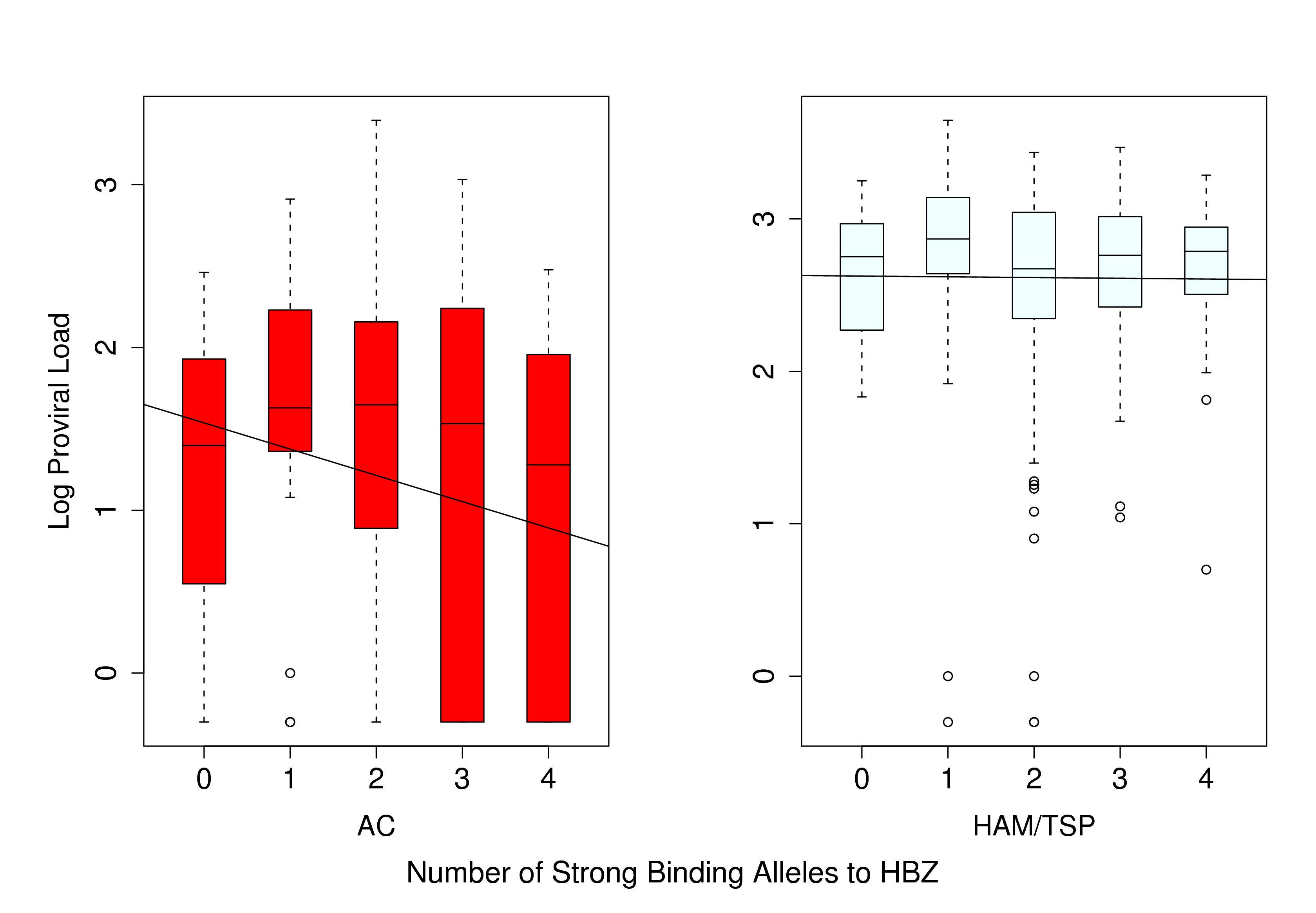


**Figure S3:** The count of strong binding alleles to HBZ per individual, against their proviral load in both the AC and HAM/TSP groups of the Kagoshima cohort. The combined p-value was 0.1. Epitope binding predictions made using Epipred.


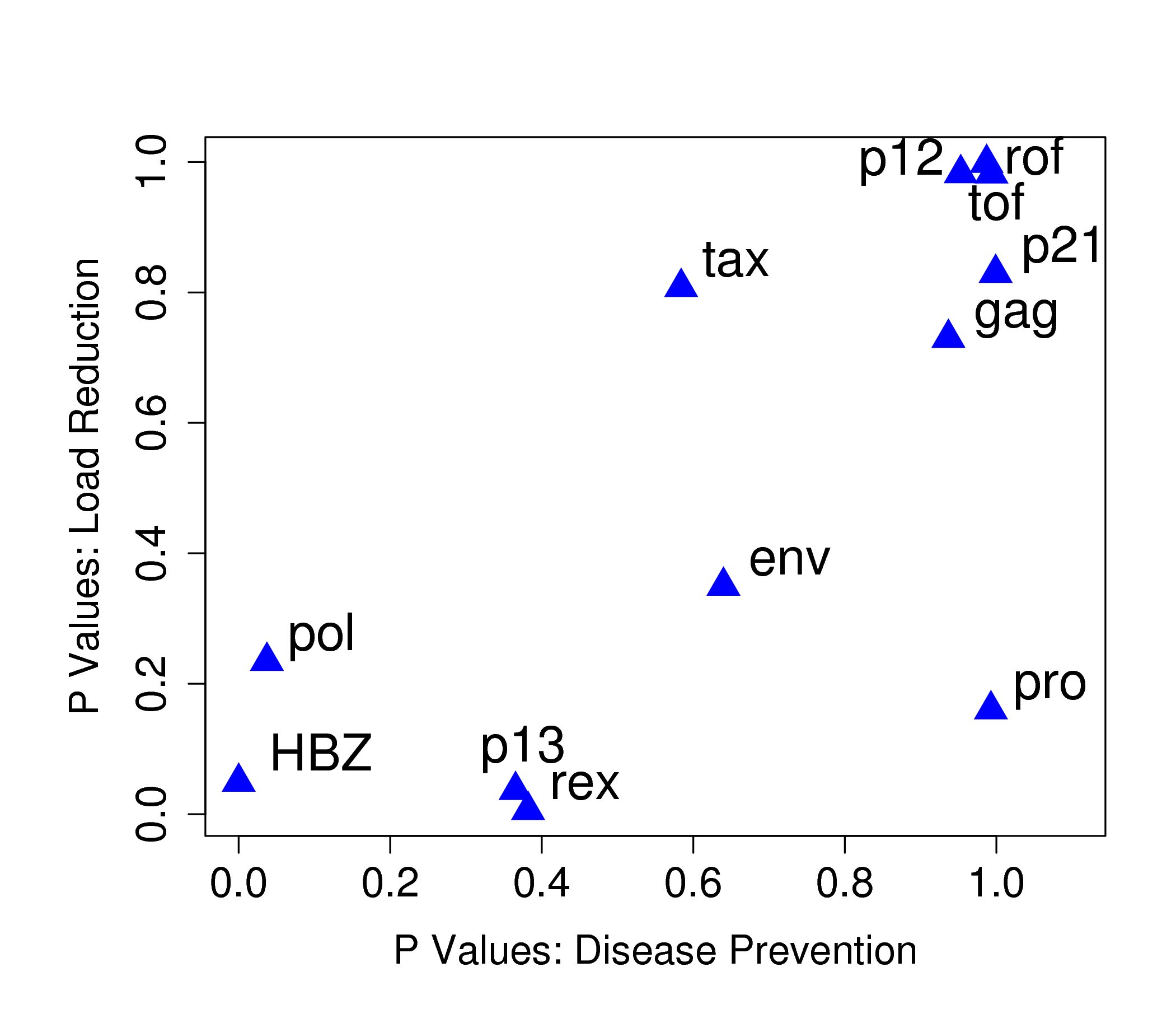


**Figure S4: HLA class I binding of peptides from different HTLV-1 proteins has a differential and correlated impact on both proviral load and HAM/TSP risk:** **Epitope binding predictions made using Epipred.** The HTLV-1 proteins were ranked according to whether they were bound significantly more strongly by asymptomatic carriers or HAM/TSP patients (x-axis). Proteins were also ranked according to whether binding their peptides was associated with a lower proviral load (y-axis). These two sets of ranks were positively correlated (R_S_=0.66, p=0.02). That is, proteins whose peptides are bound by asymptomatic carriers (left hand side of the graph) are, independently, those associated with a lower proviral load when bound (bottom of the graph).

**HTLV-I Reference Strain**

>Gag

MGQIFSRSASPIPRPPRGLAAHHWLNFLQAAYRLEPGPSSYDFHQLKKFLKIALETPARICPINYSLLASLLPKGYPGRVNEILHILIQTQAQIPSRPAPPPPSSPTHDPPDSDPQIPPPYVEPTAPQVLPVMHPHGAPPNHRPWQMKDLQAIKQEVSQAAPGSPQFMQTIRLAVQQFDPTAKDLQDLLQYLCSSLVASLHHQQLDSLISEAETRGITGYNPLAGPLRVQANNPQQQGLRREYQQLWLAAFAALPGSAKDPSWASILQGLEEPYHAFVERLNIALDNGLPEGTPKDPILRSLAYSNANKECQKLLQARGHTNSPLGDMLRACQTWTPKDKTKVLVVQPKKPPPNQPCFRCGKAGHWSRDCTQPRPPPGPCPLCQDPTHWKRDCPRLKPTIPEPEPEEDALLLDLPADIPHPKNFIGGEV

>Env

MGKFLATLILFFQFCPLIFGDYSPSCCTLTIGVSSYHSKPCNPAQPVCSWTLDLLALSADQALQPPCPNLVSYSSYHATYSLYLFPHWTKKPNRNGGGYYSASYSDPCSLKCPYLGCQSWTCPYTGAVSSPYWKFQHDVNFTQEVSRLNINLHFSKCGFPFSLLVDAPGYDPIWFLNTEPSQLPPTAPPLLPHSNLDHILEPSIPWKSKLLTLVQLTLQSTNYTCIVCIDRASLSTWHVLYSPNVSVPSSSSTPLLYPSLALPAPHLTLPFNWTHCFDPQIQAIVSSPCHNSLILPPFSLSPVPTLGSRSRRAVPVAVWLVSALAMGAGVAGGITGSMSLASGKSLLHEVDKDISQLTQAIVKNHKNLLKIAQYAAQNRRGLDLLFWEQGGLCKALQEQCRFPNITNSHVPILQERPPLENRVLTGWGLNWDLGLSQWAREALQTGITLVALLLLVILAGPCILRQLRHLPSRVRYPHYSLIKPESSL

>Pro

HPTPKKLHRGGGLTSPPTLQQVLPNQDPASILPVIPLDPARRPVIKAQVDTQTSHPKTIEALLDTGADMTVLPIALFSSNTPLKNTSVLGAGGQTQDHFKLTSLPVLIRLPFRTTPIVLTSCLVDTKNNWAIIGRDALQQCQGVLYLPEAKRPPVILPIQAPAVLGLEHLPRPPEISQFPLNQNASRPCNTWSGRPWRQAISNPTPGQGITQYSQLKRPMEPGDSSTTCGPLTL

>Pol

GKKAACNLANTGASRPWARTPPKAPRNQPVPFKPERLQALQHLVRKALEAGHIEPYTGPGNNPVFPVKKANGTWRFIHDLRATNSLTIDLSSSSPGPPDLSSLPTTLAHLQTIDLRDAFFQIPLPKQFQPYFAFTVPQQCNYGPGTRYAWKVLPQGFKNSPTLFEMQLAHILQPIRQAFPQCTILQYMDDILLASPSHEDLLLLSEATMASLISHGLPVSENKTQQTPGTIKFLGQIISPNHLTYDAVPTVPIRSRWALPELQALLGEIQWVSKGTPTLRQPLHSLYCALQRHTDPRDQIYLNPSQVQSLVQLRQALSQNCRSRLVQTLPLLGAIMLTLTGTTTVVFQSKEQWPLVWLHAPLPHTSQCPWGQLLASAVLLLDKYTLQSYGLLCQTIHHNISTQTFNQFIQTSDHPSVPILLHHSHRFKNLGAQTGELWNTFLKTAAPLAPVKALMPVFTLSPVIINTAPCLFSDGSTSRAAYILWDKQILSQRSFPLPPPHKSAQRAELLGLLHGLSSARSWRCLNIFLDSKYLYHYLRTLALGTFQGRSSQAPFQALLPRLLSRKVVYLHHVRSHTNLPDPISRLNALTDALLITPVLQLSPAELHSFTHCGQTALTLQGATTTEASNILRSCHACRGGNPQHQMPRGHIRRGLLPNHIWQGDITHFKYKNTLYRLHVWVDTFSGAISATQKRKETSSEAISSLLQAIAHLGKPSYINTDNGPAYISQDFLNMCTSLAIRHTTHVPYNPTSSGLVERSNGILKTLLYKYFTDKPDLPMDNALSIALWTINHLNVLTNCHKTRWQLHHSPRLQPIPETRSLSNKQTHWYYFKLPGLNSRQWKGPQEALQEAAGAALIPVSASSAQWIPWRLLKRAACPRPVGGPADPKEKDLQHHG

>Rof

MPKTRRRPRRSQRKRPPTPWQLPPFSLQGLHLAFQLSSIAINPQLLHFFFPSTMLFRLLSPLSPLALTALLLFLLPPSDVSGLLLRPPPAPCLLLFLPFQILSGLLFLLFLPLFFSLPLLLSPSLPITMRFPARWRFLPWRAPSQPAAAFLF

>P12

MLFRLLSPLSPLALTALLLFLLPPSDVSGLLLRPPPAPCLLLFLPFQILSGLLFLLFLPLFFSLPLLLSPSLPITMRFPARWRFLPWRAPSQPAAAFLF

>Tof

MALCCFAFSAPCLHLRSRRSCSSCFLLATSAAFFSARLLRRAFSSSFLFKYSAVCFSSSFSRSFFRFLFSSARRCRSRCVSPRGGAFSPGGPRRSRPRLSSSKDSKPSSTASSSSLSFNSSSKDNSPSTNSSTSRSSGHDTGKHRNSPADTKLTMLIISPLPRVWTESSFRIPSLRVWRLCTRRLVPHLWGTMFGPPTSSRPTGHLSRASDHLGPHRWTRYRLSSTVPYPSTPLLPHPENL

>P13

MLIISPLPRVWTESSFRIPSLRVWRLCTRRLVPHLWGTMFGPPTSSRPTGHLSRASDHLGPHRWTRYRLSSTVPYPSTPLLPHPENL

>Rex

MPKTRRRPRRSQRKRPPTPWPTSQGLDRVFFSDTQSTCLETVYKATGAPSLGDYVRPAYIVTPYWPPVQSIRSPGTPSMDALSAQLYSSLSLDSPPSPPREPLRPSRSLPRQSLIQPPTFHPPSSRPCANTPPSEMDTWNPPLGSTSQPCLFQTPDSGPKTCTPSGEAPLSACTSTSFPPPSPGPSCPT

>P21

MDALSAQLYSSLSLDSPPSPPREPLRPSRSLPRQSLIQPPTFHPPSSRPCANTPPSEMDTWNPPLGSTSQPCLFQTPDSGPKTCTPSGEAPLSACTSTSFPPPSPGPSCPT

>Tax

MAHFPGFGQSLLFGYPVYVFGDCVQGDWCPISGGLCSARLHRHALLATCPEHQITWDPIDGRVIGSALQFLIPRLPSFPTQRTSKTLKVLTPPITHTTPNIPPSFLQAMRKYSPFRNGYMEPTLGQHLPTLSFPDPGLRPQNLYTLWGGSVVCMYLYQLSPPITWPLLPHVIFCHPGQLGAFLTNVPYKRIEELLYKISLTTGALIILPEDCLPTTLFQPARAPVTLTAWQNGLLPFHSTLTTPGLIWTFTDGTPMISGPCPKDGQPSLVLQSSSFIFHKFQTKAYHPSFLLSHGLIQYSSFHSLHLLFEEYTNIPISLLFNEKEADDNDHEPQISPGGLEPPSEKHFRETEV

>HBZ

MAASGLFRCLPVSCPEDLLVEELVDGLLSLEEELKDKEEEKAVLDGLLSLEEESRGRLRRGPPGEKAPPRGETHRDRQRRAEEKRKRKKEREKEEEKQIAEYLKRKEEEKARRRRRAEKKAADVARRKQEEQERRERKWRQGAEKAKQHSARKEKMQELGIDGYTRQLEGEVESLEAERRKLLQEKEDLMGEVNYWQGRLEAMWLQ

**Supporting Information Results: protective v detrimental alleles.**

In our original analysis we compared the strength of binding of the protective alleles (A*0201 and Cw*08) with the detrimental allele (B*5401) to each of the 12 HTLV-I proteins by calculating the rank of the top 8 binding peptides from each protein to each allele and then, for each protein, using the Wilcoxon-Mann-Whitney test to compare the ranks (null hypothesis: there is no difference in the strength of binding of protective and detrimental alleles to this protein). We repeated this test using top 5 and top 10 as well as top 8 peptides. The resulting p values are in the table below. In all cases it is clear that the result for HBZ is highly statistically significant and our conclusion that protective alleles prefer to bind HBZ is robust to the exact number of ranked peptides considered.

| **Protein** | **Top 5** | **Top 8** | **Top 10** |
| --- | --- | --- | --- |
| pol | 0.90191 | 0.00050 | 0.32165 |
| env | 0.53993 | 0.00190 | 0.40296 |
| rof | 0.32675 | 0.00230 | 0.15268 |
| tax | 1.00000 | 0.33200 | 0.45437 |
| p12 | 0.32675 | 0.01680 | 0.29098 |
| rex | 0.85914 | 0.47060 | 0.35037 |
| **HBZ** | **0.00266** | **0.00020** | **0.00003** |
| gag | 0.29743 | 0.00110 | 0.24362 |
| pro | 0.01265 | 0.09700 | 0.02442 |
| tof | 0.00466 | 0.41110 | 0.01858 |
| p13 | 0.43956 | 0.85240 | 0.03864 |
| p21 | 0.16450 | 0.03410 | 0.11963 |

1. This test used predictions from both Metaserver and Epipred. Raw score could not be used in this test as the predicted binding affinity scores of Metaserver and Epipred are in different units. [↑](#footnote-ref-2)
